# Virome Analysis of Aphid Populations That Infest the Barley Field: The Discovery of Two Novel Groups of Nege/Kita-Like Viruses and Other Novel RNA Viruses

**DOI:** 10.3389/fmicb.2020.00509

**Published:** 2020-04-07

**Authors:** Hideki Kondo, Miki Fujita, Hiroshi Hisano, Kiwamu Hyodo, Ida Bagus Andika, Nobuhiro Suzuki

**Affiliations:** ^1^Institute of Plant Science and Resources (IPSR), Okayama University, Kurashiki, Japan; ^2^College of Plant Health and Medicine, Qingdao Agricultural University, Qingdao, China

**Keywords:** negevirus, kitavirus, aphid, virome, RNA seq, barley, diversity, horizontal transmission

## Abstract

Aphids (order Hemiptera) are important insect pests of crops and are also vectors of many plant viruses. However, little is known about aphid-infecting viruses, particularly their diversity and relationship to plant viruses. To investigate the aphid viromes, we performed deep sequencing analyses of the aphid transcriptomes from infested barley plants in a field in Japan. We discovered virus-like sequences related to nege/kita-, flavi-, tombus-, phenui-, mononega-, narna-, chryso-, partiti-, and luteoviruses. Using RT-PCR and sequence analyses, we determined almost complete sequences of seven nege/kitavirus-like virus genomes; one of which was a variant of the Wuhan house centipede virus (WHCV-1). The other six seem to belong to four novel viruses distantly related to Wuhan insect virus 9 (WhIV-9) or Hubei nege-like virus 4 (HVLV-4). We designated the four viruses as barley aphid RNA virus 1 to 4 (BARV-1 to -4). Moreover, some nege/kitavirus-like sequences were found by searches on the transcriptome shotgun assembly (TSA) libraries of arthropods and plants. Phylogenetic analyses showed that BARV-1 forms a clade with WHCV-1 and HVLV-4, whereas BARV-2 to -4 clustered with WhIV-9 and an aphid virus, Aphis glycines virus 3. Both virus groups (tentatively designated as Centivirus and Aphiglyvirus, respectively), together with arthropod virus-like TSAs, fill the phylogenetic gaps between the negeviruses and kitaviruses lineages. We also characterized the flavi/jingmen-like and tombus-like virus sequences as well as other RNA viruses, including six putative novel viruses, designated as barley aphid RNA viruses 5 to 10. Interestingly, we also discovered that some aphid-associated viruses, including nege/kita-like viruses, were present in different aphid species, raising a speculation that these viruses might be distributed across different aphid species with plants being the reservoirs. This study provides novel information on the diversity and spread of nege/kitavirus-related viruses and other RNA viruses that are associated with aphids.

## Introduction

Aphids (order Hemiptera: superfamily Aphidoidae) are phloem feeders and important insect pests that affect a wide range of crops. Aphid infestation can reduce both the yield and quality of crops by direct feeding and transmitting symptomatic plant viruses (Powell et al., 2006). Many aphid species act as vectors for a number of agriculturally important plant viruses (Ng and Perry, 2004; Brault et al., 2010). Most aphid-vectored plant viruses do not replicate within the aphids and are transmitted by either a non-circulative (non-persistent) or a circulative (persistent) manner (Ng and Perry, 2004; Whitfield et al., 2015; Dader et al., 2017); however, some plant rhabdoviruses (negative-sense RNA genome, family *Rhabdoviridae*) are transmitted by aphids through the circulative (persistent) manner and replicate within their vectors (Whitfield et al., 2018).

In cereal crops (within the family Poaceae), such as wheat (*Triticum aestivum* L.) and barley (*Hordeum vulgare* L.), aphids can cause great yield losses by directly feeding on the phloem sap and more significantly by transmitting several yellow dwarf disease-causing viruses (Jarosova et al., 2013; Krueger et al., 2013), such as barley yellow dwarf, cereal yellow dwarf and wheat yellow dwarf viruses (positive-sense RNA viruses, genera *Luteovirus* and *Polerovirus*, family *Luteoviridae*) (King et al., 2011). These viruses are transmitted in a circulative (persistent) and non-propagative manner by common aphid species that infest cereals, such as the bird cherry-oat aphid (*Rhopalosiphum padi*), corn leaf aphid (*R. maidis*), greenbugs (*Schizaphis graminum*), English grain aphid (*Sitobion avenae*), and rose-grain aphid (*Metopolophium dirhodum*) (Miller et al., 2002; Parry et al., 2012).

In addition to harboring numerous plant viruses, aphids are known to host several insect-specific single stranded DNA viruses [Myzus persicae densovirus (MpDNV) and Dysaphis plantaginea densovirus (DplDNV)] (van Munster et al., 2003; Ryabov et al., 2009) and positive-sense RNA viruses, the latter of which includes three picornaviruses: the Rhopalosiphum padi virus (RhPV) and aphid lethal paralysis virus (ALPV), both of the family *Dicistroviridae*, and the Brevicoryne brassicae virus (BrBV) of the family *Iflaviridae* (Moon et al., 1998; van Munster et al., 2002; Ryabov, 2007); and two unclassified RNA viruses: rosy apple aphid virus (a calici-like virus) and *Acyrthosiphon pisum* virus (APV) (distantly related to the members of the family *Solinviviridae*) (vandenHeuvel et al., 1997; Ryabov et al., 2009). Many of these viruses were discovered because of their phenotype alternations of the host aphids (Teixeira et al., 2016). More recently, analyses using next-generation sequencing (NGS) have identified several novel aphid-infecting viruses, including six positive-sense RNA viruses: three flavi-like viruses [Macrosiphum euphorbiae virus 1 (MeV-1); two jingmenviruses, Wuhan aphid virus 1 and 2 (WhAV-1 and -2)] (Shi et al., 2016b; Teixeira et al., 2016), a tetra-like virus (Aphis glycine virus 2), a nege-like virus [Aphis glycine virus 3 (ApGlV-3)] (Feng et al., 2017) and a sobemo-like virus (Macrosiphum euphorbiae virus 3) (Teixeira et al., 2018); a negative-sense RNA phlebo-like virus (Aphis citricidus bunyavirus) (Zhang et al., 2019); and a DNA densovirus (Macrosiphum euphorbiae virus 2) (Teixeira et al., 2018). Nevertheless, little is known about the viromes of aphid populations, particularly those that infest cereal plants in the field.

During the past decades, an increasing number of the novel “insect–infecting viruses” have been identified from mosquitoes (order Diptera). These viruses have a host range that is restricted to insects and are related to human arthropod-borne viral pathogens (so called arboviruses), such as dengue, Zika and West Nile viruses (family *Flaviviridae*), chikungunya virus (family *Togaviridae*) and Rift Valley fever virus (family *Phenuiviridae*) (Bolling et al., 2015). One of these insect–infecting virus groups has been proposed as the taxon “Negevirus,” consisting of alpha-like viruses from mosquitos and sandflies (order Diptera) (Vasilakis et al., 2013; Vasilakis and Tesh, 2015). Negevirus particles are likely “spherical or elliptical” in shape with diameter of 45–55 nm (Vasilakis et al., 2013; Nabeshima et al., 2014; Kawakami et al., 2016; O’Brien et al., 2017; Zhao et al., 2019). Their genomes consist of a non-segmented, positive-sense RNA, approximately 9 to 10 kb in length and comprises three open reading frames (ORF). The second and third ORFs encode two structural proteins, a predicted glycoprotein (ORF2), and a predicted membrane protein SP24 (ORF3) (Kuchibhatla et al., 2014; Fujita et al., 2017; Zhao et al., 2019; Colmant et al., 2020). Based on phylogenetic analysis, this taxon could be separated into two groups at the genus level, namely “Nelorpivirus” and “Sandewavirus” (Kallies et al., 2014). Negeviruses are distantly related to members of three plant virus genera, *Cileviru*s, *Higrevirus*, and *Blunervirus* (Vasilakis et al., 2013; Kallies et al., 2014; Nunes et al., 2017; Ramos-González et al., 2020). These genera have recently been assigned as the members of the family *Kitaviridae* (Walker et al., 2019). Kitaviruses have bi-, tri- or tetra-partite positive-sense RNA genomes (Locali-Fabris et al., 2006; Melzer et al., 2013; Hao et al., 2018), and some of them, such as citrus leprosis virus cytoplasmic type (CiLV-C, a cilevirus, which is prevalent in several countries of the American continent), have non-enveloped bacilliform particles and are transmitted by false spider mites, *Brevipalpus* spp. (class Arachnida) (Tassi et al., 2017; Freitas-Astua et al., 2018). Recently, a meta-transcriptomic approach on invertebrates expanded the diversity of nege- and nege-like viruses infecting or associated with mosquitos and flies (Webster et al., 2015, 2016; Shi et al., 2017; Medd et al., 2018; Sadeghi et al., 2018; Pettersson et al., 2019) and other invertebrates (Shi et al., 2016a; Debat, 2017; Feng et al., 2017; Nunes et al., 2017; Schoonvaere et al., 2018; Kondo et al., 2019). Nevertheless, there is still very limited information about the relationships between negeviruses and kitaviruses and also the evolutionary history of these two virus groups.

In this study, a meta-transcriptomic approach was used to investigate the viromes of aphid populations collected from a barley field for three consecutive years, 2016–2018. We identified at least 60 virus-like sequence contigs related to RNA viruses such as nege/kita-, flavi/jingmen-, tombus-, phenui- and luteoviruses, including eight putative novel RNA viruses. In particular, our study reveals the new nege/kita-like virus lineages with the members that are associated with aphid species and, thus, enhances our knowledge on the diversity and evolution of insect viruses that are related to plant viral pathogens.

## Materials and Methods

### Collection of Aphids and Species Identification

Collection of aphids were performed in the experimental field at the Institute of Plant Science and Resources (IPSR) in Okayama University, Kurashiki, Japan (34°59’ N and 133°77’ E) on April and May in 2016–2018. Aphids (total 15 aphid samples) were obtained from the colonies that infest leaves and ears of barley cultivar “Golden Promise”, “Morex”, “Kikai Hadaka”, “Minori Mugi”, “Ryofu”, “Kobin Katagi”, and three landraces (OUK327, OUU094, and OUU659), and then aphids samples were stored at −80°C until further analysis.

To determine the aphid species, a 658-bp fragment of mitochondrial DNA from the 5′ region of the mitochondrial cytochrome c oxidase subunit 1 (COI) gene, was analyzed (Foottit et al., 2008). Total genomic DNA was extracted from a portion of each aphid sample by using DNeasy Blood and Tissue Kit (Qiagen, Venlo, Netherlands). The COI sequence of mitochondrial DNA was amplified by PCR using QuickTaq HS Dye Mix (Toyobo, Osaka, Japan) with the primer pair “LepF and LepR” (Supplementary Table S1; Foottit et al., 2008). PCR fragments were purified using the Wizard SV Gel and PCR Clean-Up System (Promega, United States) and then sequenced using the conventional Sanger sequencing method with an ABI3100 DNA sequencer (Applied Biosystems, Foster City, CA, United States). The amplified COI fragments were subjected to a BlastN search and Neighbor Joining (NJ) tree construction (see below).

### RNA Extraction and RT-PCR

Total RNA from each aphid sample (pools of roughly 20–40 individual aphids from each colony) was extracted using TaKaRa RNAiso Plus Reagent (TaKaRa Biotech. Co., Shiga, Japan), following the manufacturer’s instructions. The total RNA fraction was analyzed by electrophoresis in a 1% (W/V) agarose gel in 1 × TAE buffer. For reverse transcription (RT)-PCR to detect virus-like sequences, cDNAs were synthesized using MMLV reverse transcriptase (Thermo Fisher Scientific, Waltham, MA, United States) with random hexamers, following the manufacturer’s instructions. The cDNAs were then used as templates for PCR amplification with QuickTaq HS Dye Mix. The primer sets used to amplify virus-like sequences are provided in Supplementary Table S1. The 16S ribosomal RNA of aphids was used as a reference target gene for RT-PCR using a primer set “Apisum16S_F and Apisum16S_R” for *Acyrthosiphon pisum* (Supplementary Table S1; Koramutla et al., 2016). To confirm the presence of primary and secondary parasitoid wasps, we selected two primer sets “L.testa_RpL3_F and L.testa_RpL3_R” and “DcarpF and DcarpR” for amplification of the ribosomal protein L3 (RpL3) gene of a putative primary parasitoid species (*Lysiphlebus testaceipes*, Hymenoptera: Braconidae) (this study) and the 16S rRNA gene of a putative secondary parasitoid species (*Dendrocerus carpenteri*, Hymenoptera: Megaspilidae) (Supplementary Table S1; Chen et al., 2006). PCR conditions used were as follows: an initial denaturation step at 94°C for 2 min; followed by 30 or 35 cycles of: denaturing at 94°C for 10 seconds, annealing at 53°C or 55°C for 30 seconds, then extension at 72°C for 1 min, then finishing with a final extension step at 72°C for 10 min. PCR products were then purified and subjected to Sanger sequencing.

To determine the major uncertain or unknown regions of virus-like sequences, RT-PCR was performed using the specific primer sets, and PCR fragments were then sequenced using the Sanger method. Sequences of the primers used in the RT-PCR are provided listed in Supplementary Table S1 or are available upon request.

### Next-Generation Sequencing and Read Assembly

Total RNA fractions were prepared separately from 15 aphid samples (each population from a single colony) and according to the year of aphid collection, pooled into three groups: pool-1 (total 81.9 μg, RNA integrity number: RIN = 8.1) consisted of samples from 2016 (5 aphid populations, BaA1-16-1 to -5), pool-2 (total 12.4 μg, RIN = 9.1) consisted of samples from 2017 (2 aphid populations, BaA2-17-6 and -7) and pool-3 (total 36.9 μg, RIN = 9.1) consisted of samples from 2018 (8 aphid populations, BaA3-18-8 to -16) (Table 1). Each RNA sample pool was depleted of rRNA using a Ribo-Zero kit (Illumina, San Diego, CA, United States) and then used for synthesis of a cDNA library using the TruSeq RNA Sample Preparation kit v2 (Illumina). The cDNA library obtained was subjected to deep sequencing using the Illumina HiSeq 2000 or 4000 platform (Illumina, pair-end 100 bp reads). Deep-sequencing was performed by Macrogen Inc (Tokyo, Japan). Raw reads (pool-1: 58,056,740; poo1-2: 58,030,114 and pool-3: 53,125,246) were trimmed by removing the adapter sequences and *de novo* assembled using the CLC Genomics Workbench version 11 (CLC Bio-Qiagen, Aarhus, Denmark). The assembled-contigs derived from each sample pool (over 1.0 kb contigs) were subjected to local Blast searches against the viral reference sequence (RefSeq) dataset of NCBI^1^ (the *E*-value cut-off is >0.05 for local BlastN). To roughly estimate the other sequence read origins for each NGS data set, we also conducted local Blast analyses against the following genome sequences: (1) aphid (*R. padi*) transcript data obtained from AphidBase^2^; (2) insect parasitoid wasp (*M. demolitor*, a larval lepidopteran endoparasitoid) transcript data from NCBI; (3) an aphid endosymbiotic bacterium (*Buchnera aphidicola* str. Ak, a blue alfalfa aphid *Acyrthosiphon kondoi* endosymbiont, accession no. CP002645) genome sequences; and (4) *R. padi* mitochondrion (accession no. KT447631) genome sequences. Mapping of sequence reads to each virus-like contigs or gap-filling virus-like sequences determined using RT-PCR was done using the Read Mapping algorithm of the CLC Genomics Workbench.

**TABLE 1:** List of the aphid samples collected from the barley field.

Year sample no.	Aphid species^1^ (BlastN best hit)	Barley cultivar or variety^2^	NGS group
**2016**			
BaA1-16-1	*Rhopalosiphum padi*	cv. Morex	pool-1
BaA1-16-2	*Rhopalosiphum maidis*	OUU659	pool-1
BaA1-16-3	*Rhopalosiphum padi*	cv. Kikai Hadaka	pool-1
BaA1-16-4	*Sitobion avenae*	cv. Kikai Hadaka	pool-1
BaA1-16-5	*Rhopalosiphum padi*	cv. Golden Promise	pool-1
**2017**			
BaA2-17-6	*Rhopalosiphum maidis*	cv. Golden Promise	pool-2
BaA2-17-7	*Sitobion avenae*	cv. Kikai Hadaka	pool-2
**2018**			
BaA3-18-8	*Rhopalosiphum maidis*	OUK327	pool-3
BaA3-18-9	*Rhopalosiphum maidis*	OUU094	pool-3
BaA3-18-10	*Rhopalosiphum maidis*	cv. Minori Mugi	pool-3
BaA3-18-11	*Rhopalosiphum maidis*	cv. Minori Mugi	pool-3
BaA3-18-12	*Rhopalosiphum maidis*	cv. Kobin Katagi	pool-3
BaA3-18-13	*Rhopalosiphum maidis*	cv. Kobin Katagi	pool-3
BaA3-18-14	*Rhopalosiphum maidis*	cv. Ryofu	pool-3
BaA3-18-15	*Rhopalosiphum maidis*	cv. Golden Promise	pool-3

*^1^The species of the aphid colonies were identified by sequencing the nucleotides of fragments of a mitochondrial gene cytochrome c oxidase 1 (COI) using RT-PCR. Rhopalosiphum padi (the bird cherry-oat aphid); R. maidis (the corn leaf aphid); Sitobion avenae (the English grain aphid). ^2^The five aphid samples (BaA3-18-10 to BaA3-18-14) were obtained from the barley plants grown in a different area of the IPSR field.*

### Sequence Analysis and Database Search

Open reading frames were identified using Enzyme X v3.3.3^3^. Pairwise sequence comparisons were performed using the Sequence Demarcation Tool version 1.2, with the MUSCLE alignment (Muhire et al., 2014). The conserved protein domains were searched using the NCBI conserved domain database^4^. Putative transmembrane domains were predicted using the TMHMM server version 2.04^5^ (Krogh et al., 2001). Blast or reverse Blast searches were run on the non-redundant (nr) DNA and protein databases from NCBI (nucleotide collection, nr/nt; transcriptome shotgun assembly, TSA).

### Phylogenetic Analyses

Maximum-likelihood (ML) tree construction was carried out as described previously with minor modifications (Kondo et al., 2015, 2016). Multiple amino acid alignments were obtained by using MAFFT online version 7^6^, set to the default parameters (Katoh and Standley, 2013). Ambiguous portions of the alignment were removed using Gblocks online version 0.91b^7^ with the stringency levels lowered for all parameters (Talavera and Castresana, 2007). ML trees were then generated using the PhyML 3.0 online program^8^ with automatic model selection by Smart Model Selection (Guindon et al., 2010; Lefort et al., 2017). The NJ trees (Saitou and Nei, 1987) were constructed based on the multiple alignments using MAFFT. Then the phylogenetic trees were refined using FigTree version 1.3.1 software^9^.

## Results and Discussion

### Identification of Aphid Species Collected From the Barley Field

In April and May in 2016–2018, we collected a total of 15 aphid population samples from the colonies on the barley plants that were grown in the experimental field of IPSR, Okayama (Table 1). Analysis of the mitochondrial COI sequence using genomic PCR and sequencing indicated that the aphid species collected were *R. padi* (the bird cherry-oat aphid), *R. maidis* (the corn leaf aphid), and *S. avenae* (the English grain aphid) (Table 1 and Supplementary Figure S1 and data not shown). *R. maidis* may be the predominant species of aphid populations in this barley field because *R. maidis* was obtained during each year of sampling and was also the main species collected of the aphid populations (10 out of 15 samples) (Table 1). Nevertheless, further intensive surveys are required to determine the actual aphid population and their dynamics in the field.

### Aphid Transcriptomic Analysis Using NGS

**FIGURE 1:**
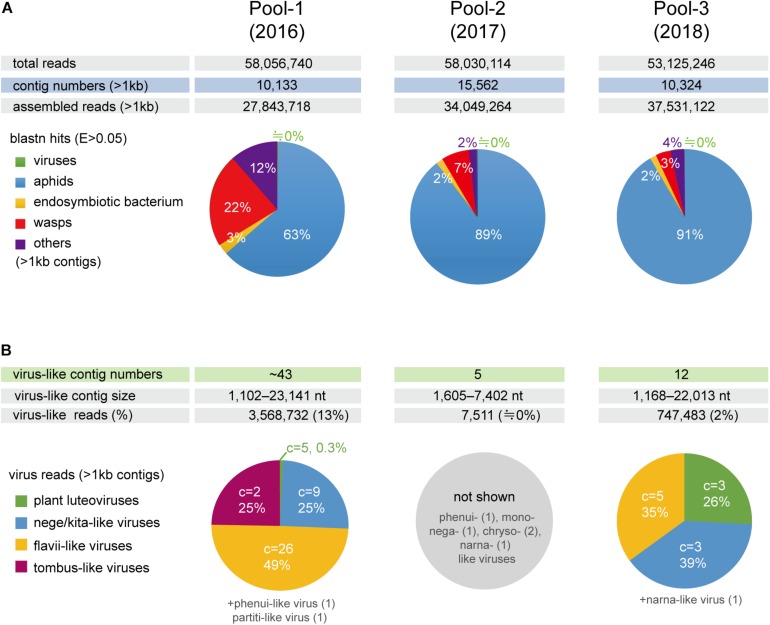
Number of *de novo-*assembled contig sequences in the three libraries (pools 1–3) derived from the barley aphid transcriptomic analysis using NGS. **(A)** Distribution of the contigs based on organisms (aphids, parasitoid wasps, endosymbiont bacterium, viruses, and others) analyzed using BlastN matches with an *e*-Value < 0.05 for the selected reference sequence data (transcriptomic or genomic sequences) (see Section “Materials and Methods”). **(B)** The number of raw reads mapping to virus-like contig sequences related to different viral groups (families or proposed taxa). Charts show the percentage of raw read, estimated using a read mapping approach, according to the virus groups in each library, except for pool-2, where only a small number of virus-like contigs were identified. C = the number of virus-like contigs.

To investigate the virome of these aphid samples, total RNA samples were pooled according to the year of sampling (pool-1 to pool-3) and subjected to transcriptomic analysis using NGS. The *de novo* assembled contigs that were larger than 1.0 kb (pool-1: 10,133; pool-2: 15,562; and pool-3: 10,324 contigs) were subjected to local Blast (tBlastX) analyses (Figure 1A). Most of the contigs were associated with aphids (63–91% contigs), with much fewer portions being associated with wasps (3–22%) and endosymbionts (2 or 3%). Aphid samples of the pool-1 might be highly parasitized by parasitoids (22% contigs), such as *L. testaceipes* compared with those of other pools (7% or 3%) (Figure 1A and data not shown). Among the total contigs (>1 kb), at least 60 contigs (pool-1: ∼43 contigs; pool-2: 5 contigs; and pool-3: 12 contigs) were virus-related sequences, with the length ranging from 1,102 to 23,141 nt (Figures 1A,B and see below). The proportions of sequence reads related to viral sequences largely differed among three libraries; 13%, 0%, and 2% for pool-1, pool-2, and pool-3, respectively (Figure 1B).

### Identification of Virus-Related Sequences From the Aphid Transcriptomes

Local Blast analyses revealed that the virus-like contigs present in the aphid transcriptomes have sequence similarity to relatives or members of the virus families, the *Kitaviridae* (or proposed Nelorpivirus and Sandewavirus), *Flaviviridae*, *Tombusviridae*, *Phenuiviridae*, *Narnaviridae*, *Chrysoviridae*, *Partitiviridae*, *Rhabdoviridae*, and *Luteoviridae* (Figure 1B and Table 2). Notably, virus-like reads were predominantly mapped to virus-like contigs associated with nege/kitaviruses (25%), flavi/jingmenviruses (49%), and tombus-like virus (25%) in pool-1; and nege/kitaviruses (39%), flavi/jingmenviruses (35%), and luteoviruses (26%) in pool-3 (Figure 1B). The three luteovirus-like contig sequences in pools 1 and 3 are likely derived from plant viruses including barley yellow dwarf virus (a well-known luteovirus, family *Luteoviridae*). The detailed analysis of luteovirus, or luteo-like virus sequences identified in the current study will be reported elsewhere as a part of the ongoing barley virome project conducted in this barley field.

**TABLE 2:** Major virus-like sequences from the barley aphid transcriptomes.

Contig name^1^	Size (nt)	Read no.	Virus or tentative^2^ virus name	Aphid^3^	Accession no.
**Nege/kita-like virus sequences**					
BaA1_c12	10,318	328,424	Wuhan house centipede virus 1	Rp, Sa	LC516834
BaA3_c89^4a^	8,890	253,596	barley aphid RNA virus 1	Rm	LC516835
BaA1_c637^ 4a^	6,421	1,390	barley aphid RNA virus 1	Rp, Sa	
BaA3_c1889	9,398	22,346	barley aphid RNA virus 2	Rm	LC516836
BaA3_c133^4b^	9,321	18,472	barley aphid RNA virus 3	Rm	LC516837
BaA1_c42/165^4b,5^	9,322	303,799	barley aphid RNA virus 3	Rp, Rm, Sa	
BaA1_c14/63^5^	8,395	283,449	barley aphid RNA virus 4	Rp,	LC516838
**Flavi-like virus sequences**					
BaA3_c535	3,140	43,197	Wuhan aphid virus 1 S1	Rp, Rm, Sa	LC516839
BaA3_c172	1,168	57,745	Wuhan aphid virus 1 S2		LC516840
BaA3_c296	2,839	77,321	Wuhan aphid virus 1 S3		LC516841
BaA3_c581	2,758	77,906	Wuhan aphid virus 1 S4		LC516842
BaA1_c359	23,141	21,307	Sitobion miscanthi flavi-like virus 1	Rp, Sa	LC516843
BaA1_c652	15,288	5,199	barley aphid RNA virus 9	Rp, Sa	LC516844
BaA1_c1578	3,691	1,025	barley aphid RNA virus 9		
BaA1_c891	3,749	742	barley aphid RNA virus 9		
BaA3_c3690^ 4c^	21,997	5,333	barley aphid RNA virus 10	Rm	LC516845
BaA1_c326^4c^	21,210	18,335	barley aphid RNA virus 10	Rm	
BaA1_c33	10,551	1,440	barley aphid RNA virus 10	Rp	LC516846
BaA1_c194	5,869	877	barley aphid RNA virus 10		
**Tombus-like virus sequences**					
BaA1_c277^ 5^	3,702	198,247	Wuhan insect virus 21, S1	Rp, Sa	LC516847
BaA1_c31^ 5^	2,201	690,152	Wuhan insect virus 21, S2		LC516848
**Phenui-like virus sequences**					
BaA1_c346	7,403	2,040	barley aphid RNA virus 5	Rp, Rm	LC516849
BaA2_c4524	7,402	1,329	barley aphid RNA virus 5	Rm	LC516850
**Other insect virus-like sequences**					
BaA2_c1487	2,636	4,820	barley aphid RNA virus 6	Rm	LC516851
BaA3_c16152	2,057	239	barley aphid RNA virus 7	Rm	LC516852
BaA2_c19028	3,552	657	barley aphid RNA virus 8, S1	Rm	LC516853
BaA2_c18060	3,220	575	barley aphid RNA virus 8, S4		LC516854
BaA1_c21296	1,259	105	a partitivirus-like contig	Rm	LC516855
BaA2_c20409	1,605	127	a mononegavirus-like contig	Rm	LC516856

**FIGURE 2:**
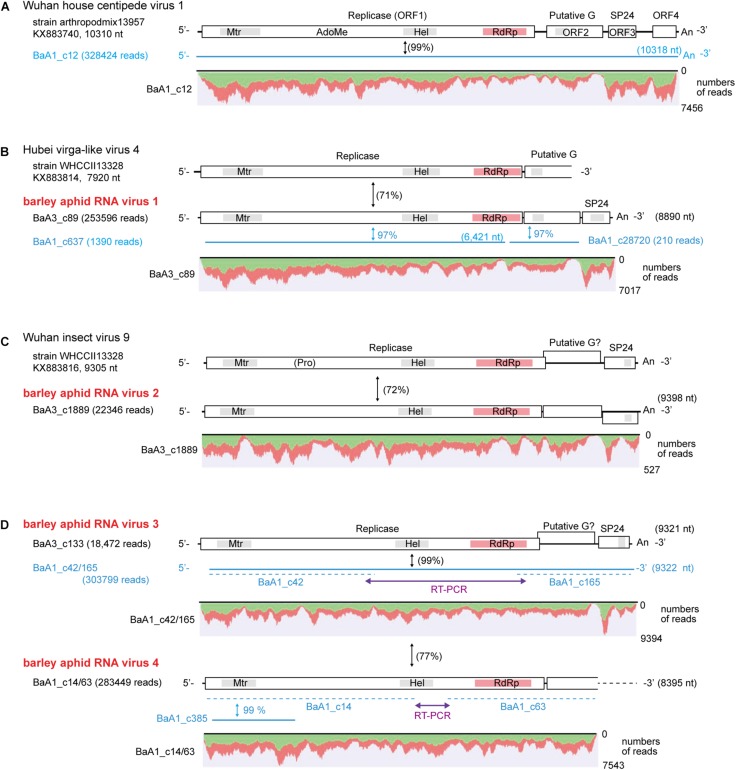
Genome organization of nege/kita-like virus sequences identified from the transcriptomes of barley aphids, *Rhopalosiphum padi; R. maidis*, and *Sitobion avenae*. **(A)** Wuhan house centipede virus 1 genome. **(B)** Hubei nege-like virus 4 genome (a reference sequence lacking the 3′ terminal part) and a related novel aphid virus (named barley aphid RNA virus 1, BARV-1) **(C)** Wuhan insect virus 9 genome (a reference sequence) and the second novel aphid virus (named barley aphid RNA virus 2, BARV-2). **(D)** The third and fourth novel aphid viruses (named barley aphid RNA virus 3 and 4, BARV-3 and -4) genome. The open boxes in the genomic RNA represent open reading frames (ORFs). The conserved domains in viral replicase are shown (methyltransferase, Mtr; FtsJ-like RNA ribosomal methyltransferase, AdoMet/FtsJ; RNA helicase, HEL; RNA-dependent RNA polymerase, RdRp). Other putative conserved domains (putative glycoprotein and SP24 protein) are indicated as gray highlights within the ORFs. Virus-like contigs identified from this study are shown as blue bars or blue lines. The sequence gaps between the two contigs for BARV-3 (BaA1_c42 and BaA1_c165) and BARV-4 (BaA1_c14 and BaA1_c63) were determined using RT-PCR and sequencing. The nucleotide sequence identities between a reference virus and a virus-like contig are shown in parentheses with black color. The nucleotide sequence identities between two virus-like contigs are shown in blue color with no parentheses. Read depth coverage throughout the assembled virus-like contigs together with two gap-filling virus-like sequences determined using RT-PCR (BaA3_c42/165 and BaA1_c14/63) is presented below the schematic structures of the viral genomes. The Y-axis shows the mapping-read coverage with the maximum read depth (read number) on each virus-like sequence. Green and red colors indicate positive and negative strand of sequence reads, respectively.

*^1^Pool-1: BaA1; Pool-2: BaA2; Pool-3: BaA3. Some other variants of virus-like contigs are shown in Supplementary Figure S7, or not shown. Plant luteovirus-related sequences are not listed here. ^2^Nucleotide and/or amino acid sequence identity of the contig sequences are shown within Table 3 and Supplementary Table S4. Ten discovered novel viruses were designated as barley aphid RNA viruses 1 to 10 (see main text). S, RNA or dsRNA segment. ^3^Aphid species in discovery colonies assessed by RT-PCR detection (see Supplementary Figure S1A). Rp: Rhopalosiphum padi; Rm, R. maidis; Sa, Sitobion avenae. ^4^These sequences are slightly different from each other at the nucleic acid level (^4a^: 3%, ^4b^: 1%, and ^4c^: 5% difference, respectively). ^5^The gap or unassembled regions were sequenced using RT-PCR (see Figure 2D and Supplementary Figure S7C).*

We performed RT-PCR to verify the presence of virus-related RNAs in the aphid samples using the specific primer sets (Supplementary Table S1). The virus targets were successfully amplified from the aphid RNA samples (Supplementary Figures S2).

### Characterization of Nege/Kita-Like Virus Sequences

We identified four contigs that resembled almost complete (coding complete) nege/kitavirus-like genome sequences (BaA1_c12; BaA3_c89; BaA3_c1889; and BaA3_c133, 8,890–10,318 nt in length) with 18,472–328,424 mapped reads (Figure 2 and Table 1). Some other smaller fragments of nege/kitavirus-like sequences were also present in pools 1 and 3, but not in pool-2 (Figure 2 and Table 1). Each of the three sets of contigs, “BaA1_c42 and BaA1_c165,” “BaA1_c14 and BaA1_c63,” and “BaA1_c637,” and “BaA1_c28720” are most likely derived from three different variants of a single viral strain, respectively (Figures 2B,D). RT-PCR and sequencing were carried out to determine their sequence gaps and, thus, semi-entire virus-like sequences were obtained (namely, BaA1_c42/165 and BaA1_c14/63, 9,322 and 8,395 nt with 303,799 and 283,449 mapped reads, respectively) (Figure 2D). In the case of contigs BaA1_c637 (1,390 reads) and BaA1_c28720 (210 reads), an attempt to connect their sequences using RT-PCR failed, possibly because of a very low level of virus titer in the samples (data not shown). These two newly assembled contigs derived from four above-mentioned virus-like sequences have large replicase-like ORFs (ORF1) followed by two putative structural protein genes for predicted glycoprotein and membrane protein SP24 (ORFs 2 and 3) (Kuchibhatla et al., 2014; Solovyev and Morozov, 2017), except for the BaA1_c12 contig, which has an additional small ORF in its 3′-proximal region, or the BaA1_c14/63 contig, which lacks the 3′-terminal region sequence (Shi et al., 2016a; Figure 2 and Supplementary Figures S3, S4).

The nucleotide sequence and encoded proteins of BaA1_c12 are almost identical (>99% nucleotide and 99–100% amino acid identities) to those of the Wuhan house centipede virus 1 (WHCV-1) (Supplementary Table S3), suggesting that the BaA1_c12 sequence is derived from a strain of WHCV-1, infecting two aphid species (*R. padi* and *S. avenae*) that infested barley plants. The two contigs BaA3_c89 and BaA1_c637 (lacking 3′-terminal region sequence) showed 97% nucleotide identity with each other and 71% nucleotide identity with Hubei virga-like virus 4 (HVLV-4) (Figure 2B and Table 3). A BlastP search revealed that the two contigs share moderate levels of amino acid sequence identity (45–58%) with HVLV-4 (Table 3). Although taxonomic criteria for species demarcation have not been established for nege-like viruses, both of the two HVLV-4-like sequences most likely represent a novel nege/kita-like virus that infects *R. padi* and *S. avenae* (BaA1_c637) or *R. maidis* (BaA3_c89) [our tentative cut-off value for the species classification criteria for nege-like viruses is <90% amino acid identities of RNA-dependent RNA polymerase (RdRp) sequences], rather than being a strain of HVLV-4; therefore we tentatively designated the virus as barley aphid RNA virus 1 (BARV-1).

**TABLE 3:** Nege/kita-like virus sequences identified from the barley aphid transcriptomes using Blast search.

Query contig	Top hit virus	QC^1^	*E*-value	identify	Accession
**BlastN search (discontiguous megablast)**					
**Query sequence: entire nucleotide sequences**					
BaA1_c12	Wuhan house centipede virus 1	99%	0.0	99%	KX883740
BaA3_c89	Hubei virga-like virus 4	52%	0.0	71%	KX883814
BaA3_c1889	Wuhan insect virus 9	99%	0.0	72%	KX883816
BaA1_c42/165	Wuhan insect virus 9	53%	0.0	72%	KX883816
BaA1_c14/63	Hubei Wuhan insect virus 9	54%	0.0	73%	KX883782
**BlastP search**					
**Query sequence: RdRp**					
BaA1_c12	Wuhan house centipede virus 1	99%	0.0	99%	APG77795
BaA3_c89	Hubei virga-like virus 4	52%	0.0	58%	APG77770
BaA3_c1889	Wuhan insect virus 9	98%	0.0	78%	APG77775
BaA3_c133	Wuhan insect virus 9	53%	0.0	57%	APG77775
BaA1_c14/63	Hubei Wuhan insect virus 9	54%	0.0	58%	APG77665
**Query sequence: p2 (putative glycoprotein)**					
BaA1_c12	Wuhan house centipede virus 1	99%	0.0	99%	APG77796
BaA3_c89	Hubei virga-like virus 4	77%	2e-112	50%	APG77771
BaA3_c1889	Wuhan insect virus 9	96%	0.0	66%	APG77776
BaA3_c133	Wuhan insect virus 9^2^	87%	3e-86	41%	APG77776
BaA1_c14/634	Wuhan insect virus 9^2^	91%	1e-92	48%	APG77776
**Query sequence: p3 (SP24 family)**					
BaA1_c12	Wuhan house centipede virus 1	99%	4e-139	100%	APG77797
BaA3_c89	Wuhan house centipede virus 1	69%	3e-46	45%	APG77797
BaA3_c1889	Hubei Wuhan insect virus 9	90%	5e-117	80%	APG77670
BaA13_c133	Hubei Wuhan insect virus 9^2^	98%	3e-68	52%	APG77670
BaA1_c14/63	not available				

*^1^QC, query coverage (%). ^2^The partial genome sequence of a Hubei Wuhan insect virus 9 variant (APG77666/APG77667) shows higher aa identity to each query sequence (59%–85%) (see Supplementary Figures S4B–E).*

Contig BaA3_c1889, derived from *R. maidis*, appeared to be a novel nege/kita-like virus related to Wuhan insect virus 9 (WhIV-9) because they share moderate levels of identities at the nucleotide (72%) and amino acid (66%–80%) sequence (Tables 2 and 3). We named this putative nege/kita-like virus as barley aphid RNA virus 2 (BARV-2). Two contigs, BaA3_c133 and BaA1_c14/63 (lacking 3′-terminal region sequence), together with BaA1_c42/165 (almost identical to the BaA3_c133 contig) also showed similar levels of nucleotide identity (72%) with WhIV-9 and its relatives, Hubei Wuhan insect virus 9 (HWIV-9) (Table 3). Their proteins showed lower levels of amino acid sequence identity (41%–58%) than those of the BaA3_c1889 contig (Table 3). In addition, they shared 77% nucleotide and 69% amino acid sequence identities between each other. Thus, contigs BaA3_c133 and BaA1_c42/165, and BaA1_c14/63 appear to represent two novel nege/kita-like viruses, which we tentatively designated as barley aphid RNA virus 3 and 4 (BARV-3 and -4), and it associated with *R. padi* (BaA1_c14/63, BARV-4); *R. maidis* (BaA3_c133, BARV-3); or *R. padi*, *R. maidis*, and *S. avenae* (BaA1_c42/165, BARV-3) (Table 2 and Supplementary Figure S2A).

### Search for Nege/Kita-Like Virus Sequences Using the TSA Database

**FIGURE 3:**
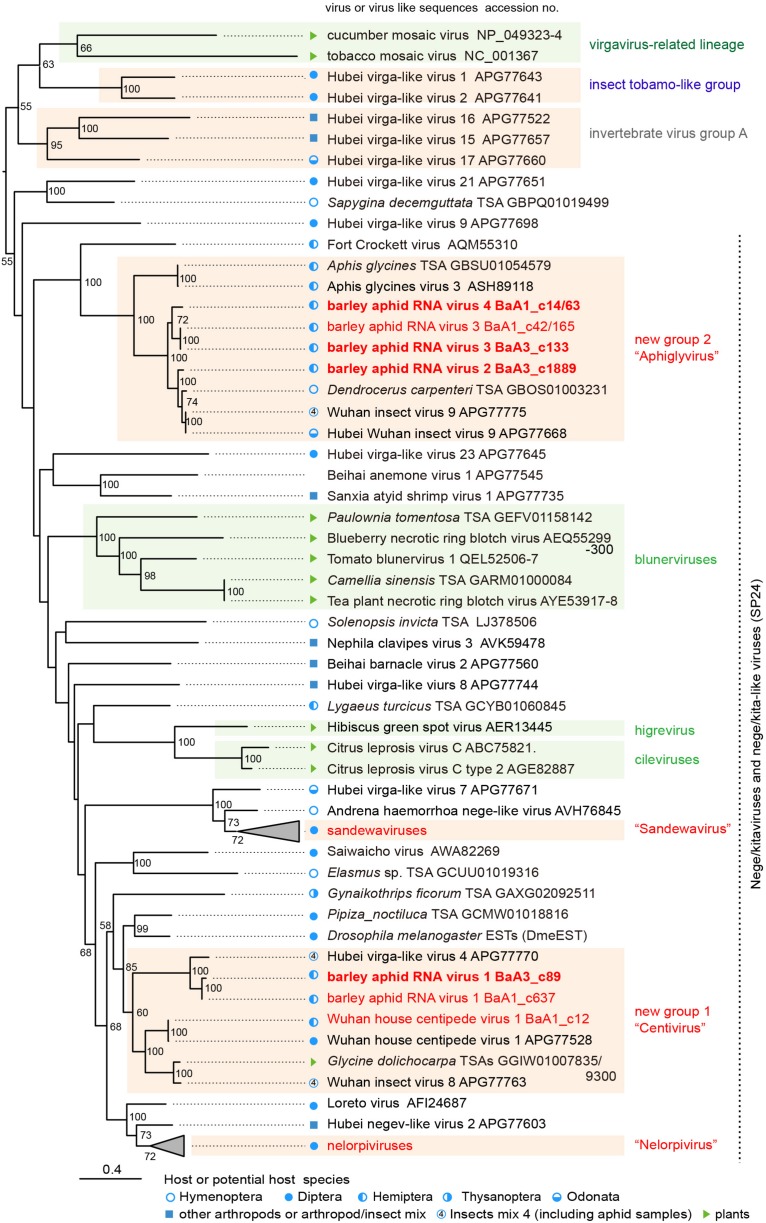
Phylogenetic relationships of the nega/kitaviruses and their related viruses and other virus-like sequences. The maximum likelihood (ML) phylogenetic tree was constructed using PhyML 3.0 based on a multiple amino acid sequence alignment of the replicase protein or its candidate sequences. A model LG + I + G + F was selected as a best-fit model for the alignment. The tree was rooted using the midpoint rooting method. Virus names referring to plant-infecting viruses (genera *Cilevirus, Higrevirus*, and *Blunervirus*, family *Kitaviridae*) and insect-infecting or associated viruses are followed by the GenBank accession numbers of their sequences. The selected members of the proposed groups “Sandewavirus” and “Nelorpivirus” are displayed as collapsed triangles (see Supplementary Table S3 for the virus names in the collapsed triangles). Nege/kitavirus-like transcriptome shotgun assembly (TSA) sequences, discovered by this study (see Supplementary Table S2) or our previous study (Kondo et al., 2019), were also included in this analysis. A library of insect mixtures (WHCCII, insect virus 4) for the meta-transcriptomic analysis included two aphid species, a parasitoid wasp, and several other insects (Shi et al., 2016a). The hosts, or possible hosts, for nege/kitaviruses or virus-like sequences present in TSAs are marked with symbols (circles, squares, and triangles, respectively). Vertical dashed lines indicate that most of those viruses or virus-like sequences potentially encode conserved small proteins (SP24 family) (Kuchibhatla et al., 2014). The selected members of distantly related virus lineage, belonging to the plant virgavirus-related lineage (tobacco mosaic and cucumber mosaic viruses), insect tobamo-like virus group (Hubel virga-like viruses 1 and 2), and invertebrate virus group A (Hubel virga-like viruses 15, 16, and 17) (Kondo et al., 2019), were used as the outgroups. The scale bar represents amino acid distances. The numbers at the nodes are bootstrap values of >50%.

Recently, the presence of several nege/kita-like viruses and related virus-like sequences, has been reported from invertebrate meta-transcriptome analyses or surveys of insect transcriptomic datasets (Shi et al., 2016a; Kondo et al., 2019). To further explore the presence of unknown nege/kitavirus-like sequences, we conducted tBlastN searches against publicly available TSA datasets using BARV-1 and BARV-2 sequences as queries. The search identified several TSA sequences derived from hemipteran, hymenopteran, and dipteran species with a few plant species that represent nearly complete (>9.0 kb lengths) or partial viral genome sequences (Supplementary Table S2 and data not shown). Two plant TAS sequences from a tree species, *Paulownia tomentosa* (family Paulowniaceae) (Fan et al., 2016), most likely represented the segments of a novel RNA virus belonging to the blunervirus-lineage (Supplementary Table S2 and also see Figure 3).

### Phylogenetic Relationships Among Nege/Kita- and Nege/Kita-Like Viruses

To understand the relationships between newly discovered nege/kita-like viruses, viral-like TSAs and reported nege/kitaviruses, we conducted ML phylogenetic analysis based on amino acid alignment of their replicase and replicase-like sequences. The ML tree showed that the BARV-1 variants clustered with HVLV-4 and its relatives, WHCV-1 and Wuhan insect virus 8 (WhIV-8) (referred to as “group 1”); whereas the BARV-2, -3, and -4 form a well-supported clade with the relatives of WhIV-9, and HWhIV-9, and ApGIV-3 from *Aphis glycines* (the soybean aphid) (referred to as “group 2”) (Figure 3). These two virus groups together with other nege/kita-like viruses and arthropod virus-like TSAs, fill the phylogenetic gaps between negevirus-related (nelorpiviruses and sandewaviruses) and kitavirus-related (cile/higreviruses and blunerviruses) lineages (Vasilakis et al., 2013; Kallies et al., 2014; Nunes et al., 2017; Figure 3 and Supplementary Table S2). Our phylogenetic analysis, thus, suggests that novel groups 1 and 2 of nege/kita-like viruses including aphid-infecting viruses belong to two new distinct genera, tentatively designated as “Centivirus” (after Wuhan house centipede virus 1) and “Aphiglyvirus” (after Aphis glycines virus 3), respectively. Members of these two proposed genera, together with two proposed taxa of arthropod-restrictive viruses, “Nelorpivirus” and “Sandewavirus,” which all have non-segmented genomes, could be classified into a novel viral family or be assigned to the family *Kitaviridae*. However, it is to too early to establish a family for these viruses or to assign them into the *Kitaviridae*, because there is no reliable statistical support for these lineages within the currently available phylogenetic trees (Kallies et al., 2014; Nunes et al., 2017; Kondo et al., 2019; Ramos-González et al., 2020; Figure 3). The result of molecular phylogenetics indicate that the two major lineages of kitaviruses (cile/higreviruses and bulunerviruses) are not monophyletic, which all have two or multi-segmented RNA genomes and separately encode their replicase (RdRp) and SP24 family protein on the different segments (Locali-Fabris et al., 2006; Melzer et al., 2013; Hao et al., 2018), suggesting that genome segmentation possibly occurred independently during the evolution of kitaviruses. As previously proposed for plant rhabdoviruses (Kondo et al., 2017), the genome segmentation event may be associated with the vector mites (*Brevipalpus* spp.), at least in the case of the cile/higreviruses lineage.

### Host Ranges and Spread of Nege/Kita-Like Viruses

WHCV-1 (a centivirus) was identified from the house centipede (*Scutigeridae* sp., phylum Arthropoda) and also from three other arthropods, spiders, and insects (WHCCII, insect mix 4) (Shi et al., 2016a). Because the last sample WHCCII included two aphid species (*Hyalopterus pruni* and *Aulacorthum magnolia*), a parasitoid wasp (*Aphelinus* sp., Hymenoptera), and some other insects such as dipterans, an orthopteran, and a coleopteran (Shi et al., 2016a), WHCV-1 may have a wide host range within arthropods (centipedes, spiders, and insects, including aphids as described in this study). Other centiviruses, WhIV-8 and HVLV-4, were also identified from the same insect pool (WHCCII) along with WhIV-9 (an aphiglyvirus), but their insect host ranges are still uncertain (Shi et al., 2016a). WHCV-1 and the two novel viruses, BARV-1 and BARV-3, were detected in four aphid colonies from the 2016 sample (WHCV-1 and BARV-1) or six aphid colonies from both 2016 and 2018 samples (BARV-3); whereas other viruses or variants were detected in a single aphid colony from the 2016 (BARV-4) and 2018 samples (BARV-2) (Supplementary Figure S2A). Notably, WHCV-1 and BARV-3 were detected in three different aphid species, *R. padi, R. maidis* and *S. avenae* collected in 2016 (Supplementary Figure S2A). Intriguingly, our preliminarily results showed the presence of these aphid virus-related sequences in the barley plant samples from the same field in the same year (HK and MF unpublishded results). Therefore, barley and/or other plants may potentially reserve aphid viruses and facilitate the horizontal transmission of the viruses among aphid populations, similarly to what was reported previously for an aphid picornavirus (RhPV) (Gildow and Darcy, 1988; Ban et al., 2007). WHCV-1-, BARV-3- and BARV-4-related sequences were identified in the TSA datasets derived from the Polish wheat (*Triticum polonicum*) (Supplementary Figure S5). WhIV-8- and BARV-2-like sequences were found in an allopolyploid bean species (*Glycine dolichocarpa*, Fabaceae) or the pomegranate (*Punica granatum*, Lythraceae) (Supplementary Figure S5 and Supplementary Table S2), and some insect-specific viruses (densoviruses and dicistroviruses) have also been found from plant materials, such as cucumber, bean, *Brassica oleracea*, red cestrum (Solanaceae), maize and sea barley (van Munster et al., 2005; François et al., 2014; Maina et al., 2017; Wamonje et al., 2017; Roumi et al., 2020). Thus, these findings support the notion that plants serve as passive vectors for the transmission of aphid viruses.

WhIV-9, an aphiglyvirus from the WHCCII insect pool, has been identified from a dragonfly (*Odonata* sp., order Odonata) (Shi et al., 2016a), whereas WhIV-9-like TSA sequences was also found in *D. carpenter*, an aphid hyperparasitoid wasp (Figure 3 and Supplementary Table S4). The presence of WhIV-9-like sequences in the hyperparasitoid wasp may suggest that the virus could be horizontally transmitted between this wasp and an aphid host through a primary parasitoid. In cereal aphids, such as *S. avenae*, the attack of hyperparasitoids against primary parasitoids has been observed in the field (Holler et al., 1993). In our meta-transcriptome analyses, some aphid populations might be infected with the primary parasitoids, although it is still unknown whether the hyperparasitoids are also present in the IPSR field (Supplementary Figure S2A). A primary parasitoid (*L. testaceipes*), but not a secondary parasitoid (*D. carpenteri*), was detected in the aphid samples using RT-PCR (Supplementary Figure S6). It has been reported that an RNA virus, Lysiphlebus fabarum virus, related to BrBV (an aphid iflavirus), was found in an aphid parasitoid wasp (*Lysiphlebus fabarum*) (Lüthi et al., 2019). Its closest related virus, Venturia canescens picorna-like virus, replicates in both a parasitoid wasp and a lepidopteran host (Reineke and Asgari, 2005). An aphid picornavirus ALPV has been detected in the honey bees and its predator hornet *Vespa velutina* (Hymenoptera) (Granberg et al., 2013; Yang et al., 2019). Therefore, further studies are necessary to understand the dynamics and spread of nege/kita-like viruses among barley aphid populations, including their parasitoid and hyperparasitoid hymenopterans.

### Characterization of Flavi-Like Virus Sequences

In addition to the nege/kita-like viruses (centi- and aphiglyviruses), we identified several contigs related to other RNA viruses, such as flavi/jingmen- and tombus-like virus sequences (Figure 1 and Table 2). WhAV-1, a jingmenvirus, which belongs to a recently identified group of the four-segmented RNA viruses related to unsegmented flaviviruses (Shi et al., 2016b; Temmam et al., 2019), was one of the major constituents of flavi/jingmenvirus-like reads (Figure 1 and Table 2). Sequence homology analyses suggest that at least three variants of WhAV-1 (1a, 1b, and 1c variants) were associated with barley aphids (Supplementary Figure S7A and Supplementary Table S4). The “1b” variant of WhAV-1 (the BaA3_c535 contig and some others), which is related to the reference viral sequence sharing ∼87% nucleotide and ∼95% amino acid sequence identities, was found in five aphids colonies containing all three species from the 2016 sample and *R. maidis* from the 2018 sample (Supplementary Figure S2B). In contrast, the “1a” (the BaA1_c139 contig) and “1c” (the BaA1_c13 contig) variants, which are related to the reference sequence sharing 91% and 78% nucleotide and 92% and 87% amino acid sequence identities, were only detected in an *R. padi* colony from the 2016 sample (BaA1_c139) and in two aphid colonies (*R. padi* and *R. maidis*) from the 2016 sample (BaA1_c13), respectively (Supplementary Figure S2B and data not shown). WhAV-1b sequences (BaA3_c535, c172, c296, and c581 for RNA segments 1–4) have been deposited into the DDBJ data bank as a representative genome sequence (Table 2). In the phylogenetic tree, based on the non-structural protein NSP1 (NS5-like, RdRp) encoded in segment 1, jingmenviruses are divided into two different clades; the WhAV-1 variants and a second aphid-associated jingmenvirus, WhAV-2, form a subclade within the clade consisting of insect and some other arthropod jingmenviruses; whereas the second clade contains jingmenviruses infecting ticks (class Arachnida), such as jingmen tick virus, which may be a novel tick-borne arbovirus that infects humans and other mammals (Jia et al., 2019; Supplementary Figure S7B). WhAV-1 sequences were identified in the aphid-free barley samples as mentioned above (HK and MF unpublishded results), and interestingly, a variant of WhAV-2 was found in a pea plant transcriptome (*Pisum sativum*, accessions MK948535–8, deposited by Y.Z.A. Gaafar and H. Ziebell) (Supplementary Figure S2B and Supplementary Table S2), raising a speculation that jingmenviruses that infect aphids may have been horizontally transmitted among aphid populations, with plants as the reservoirs (Gildow and Darcy, 1988; Ban et al., 2007).

**FIGURE 4:**
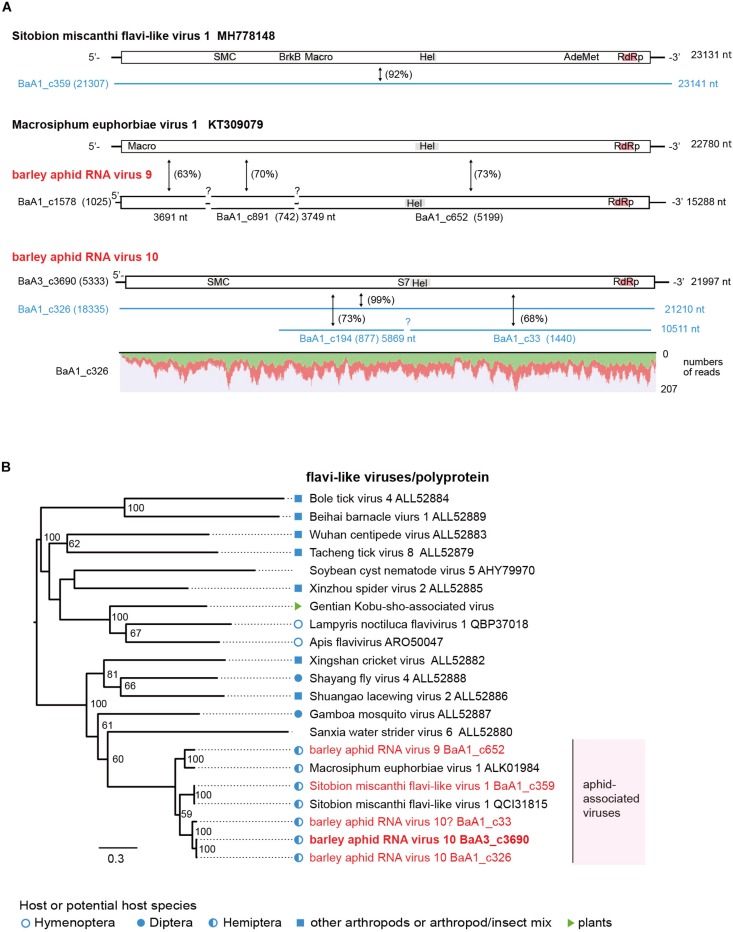
Genome organizations and phylogenetic relationships of flavi-like viruses. **(A)** The genome structures of flavi-like viruses, Sitobion miscanthi flavi-like virus 1, Macrosiphum euphorbiae virus 1, and two novel flavi-like viruses (named barley aphid RNA virus 9 and 10, BARV-9 and -10) from *Rhopalosiphum padi, R. maidis*, and *Sitobion avenae*. Conserved domains for viral polyproteins or other proteins are shown as gray or red highlights within the ORFs (Macro domain, a high-affinity ADP-ribose binding module; SMC super family, chromosome segregation ATPase; BrkB, This family may act as a virulence factor; S7, Flavivirus NS3 serine protease; HEL, RNA helicase; AdoMet, S-adenosylmethionine-dependent methyltransferases; RdRp, RNA-dependent RNA polymerase). Virus-like contigs derived from this study are represented as black bars or blue lines and other information is the same as shown in Figure 2. Read depth coverage throughout the assembled virus contig is shown below the schematic structure of the viral genome. **(B)** Phylogenetic relationships of flavi-like viruses. The ML phylogenetic tree was constructed using a multiple amino acid sequence alignment of the polyprotein or its candidate sequences. The scale bar represents amino acid distances. The numbers at the nodes are bootstrap values of >50%.

Another type of flavivirus-like sequences seems to be the minor populations among aphid flavi-related viruses (Table 2). The BaA1_c359 contig is derived from a variant Sitobion miscanthi flavi-like virus 1 (SmFLV-1, MH778148 deposited by T. Li), showing 92% nucleotide and 98% amino acid sequence identities (Supplementary Table S4). The BaA1_c652 contig (probably together with BaA1_c1578 and c891 contigs) sharing 73% nucleotide and 62% amino acid sequence identities with that of MeV-1 from European *M. euphorbiae* populations (Teixeira et al., 2016) and the BaA3_c3690 together with its two variants BaA1_c326 and c33 (a partial sequence) contigs showing 67–68% nucleotide and 44–59% amino acid sequence identities with that of SmFLV-1, are likely to represent two novel flavi-like viruses. Thus, we tentatively named barley aphid RNA virus 9 (BARV-9) and barley aphid RNA virus 10 (BARV-10) (Figure 4A and Table 2). The complete coding sequence of BARV-3 (BaA3_c3690) is 21,997 nt in length, similar to the genomes of MeV1 (22,780 nt) and SmFLV-1 (23,131 nt) (Figure 4A). The single large ORF of BARV-9 encodes a putative polyprotein of 7,081 amino acids, containing putative helicase, NS3 serine protease, and RdRp motifs, but not the motifs predicted for structural proteins and methyltransferase (Figure 4A). In the phylogenetic tree based on polyproteins (Figure 4B), aphid-associated flavi-like viruses form a clade and clustered with some invertebrate flavi-like viruses, whereas its sister clade contains several invertebrate flavi-like viruses and a plant virus, Gentian Kobu-sho-associated virus (Kobayashi et al., 2013; Bekal et al., 2014; Shi et al., 2016b; Remnant et al., 2017), that still have an uncertain taxonomic status. SmFLV-1 and BARV-9 variants were found in two aphid colonies (*R. padi* and *S. avenae*) from the 2016 sample and SmFLV-1 probably also in a *R. maidis* colony from the 2018 sample in which low levels of viral RNA accumulation was detected using RT-PCR, whereas no virus reads were found in the NGS data, thus expanding its aphid host range. BARV-9-like TSA sequences were also found in *S. avenae* (Supplementary Figure S5). BARV-10 variants were detected in two *R. maidis* colonies from the 2016 and 2018 samples (BaA1_c326) or an *R. padi* colony from the 2016 sample (BaA1_c33) (Supplementary Figure S2B). Similar to the nege/kita-like viruses, multiple flavi-like viruses belonging to at least three viruses (BARV-9, BARV-10 and SmFLV-1) were present in barley aphid populations that infest the IPSR field; however, their prevalence in the aphid populations seems to be much lower than that of nege/kita-like viruses and a jingmenvirus (WhAV-1) (Table 2 and Supplementary Figure S2).

### Characterization of Tombus-Like Virus Sequences

Recently, the diversity of the tombusvirus-like lineage has been greatly expanded by the discovery of a large number of tombus-like viruses from invertebrates (Shi et al., 2016a). One of these viruses, Wuhan insect virus 21 (WhIV-21), was detected in the WHCCII insect pool; the same pool in which the above mentioned viruses were detected. In this study, we found a variant of WhIV-21 (BaR1_c277 and c31 contigs, sharing 83% and 73% nucleotide and 90% and 50% amino acid sequence identities with WhIV-21) in *R. padi* and *S. avenae* aphids from the 2016 sample, but not from *R. maidis* (Table 2, Supplementary Figure S2B, and Supplementary Table S4). The complete coding sequences of WhIV-21 were verified using RT-PCR and sequencing (Supplementary Figure S7C). The read numbers of WhIV-21 (198,247 reads for BaR1_c277, and 690,152 reads for BaR1_c31), whose sequences were also detected in the barley samples (HK and MF unpublishded results), were higher than those of jinmenviruses and similar to some nege/kitaviruses (Table 2). The ML phylogenetic tree, based on RdRp sequences encoded by the RNA1 segments, showed that WhIV-21 is clustered with a bee pathogen, Chronic bee paralysis virus (CBPV, proposed genus “Chroparavirus”), and several invertebrate tombus-like viruses (Shi et al., 2016a; Bigot et al., 2017; Supplementary Figure S7D). Similar to CBPV, which may form a ellipsoidal virion, the WhIV-21 RNA2 segment seems to encode two proteins; a putative virion glycoprotein and SP24 homolog that probably have features of the major structural components, similar to what was previously proposed for CBPV and some negeviruses (Kuchibhatla et al., 2014; Solovyev and Morozov, 2017).

### Characterization of Other Novel Aphid-Associated Virus Sequences

**FIGURE 5:**
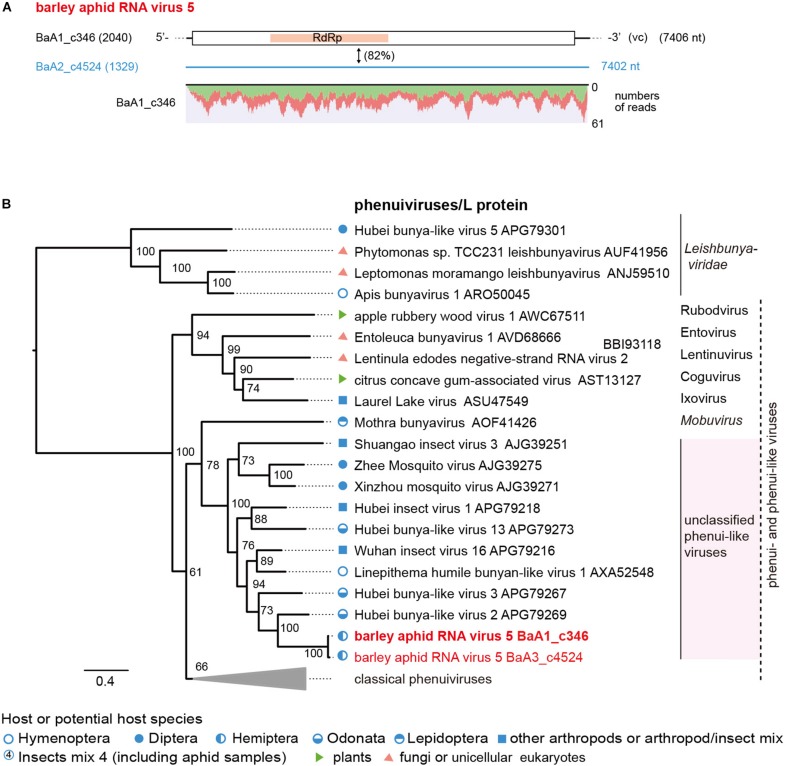
The genome organization and phylogenetic relationships of phenui- and phenui-like viruses. **(A)** The genome structure of the putative L segments of a phenui-like virus (named barley aphid RNA virus 5, BARV-5) identified from *Rhopalosiphum padi*, *R. maidis*, and *Sitobion avenae* or *R. maidis* transcriptomes. The conserved domain for viral L proteins is shown as a red highlight within the ORF (RNA-dependent RNA polymerase, RdRp). Virus-like contigs in this study are shown as a black bar or a blue line, together with their read numbers in parentheses, and the nucleotide sequence identity between two virus-like contigs is shown. Read depth coverage throughout the assembled virus contig is shown below the schematic structure of the viral genome. **(B)** Phylogenetic relationships of BARV-5 and other selected phenuiviruses (family *Phenuiviridae*) and phenui-like viruses. The ML phylogenetic tree was constructed using a multiple amino acid sequence alignment of the L proteins or its candidate sequences. The selected members of the genera within the family *Phenuiviridae* are displayed as a collapsed triangle (see Supplementary Table S3 for the virus names in the collapsed triangle). Some members of family *Leishbuniyaviridae* were used as the outgroups. The scale bar represents amino acid distances. The numbers at the nodes are bootstrap values of >50%.

**FIGURE 6:**
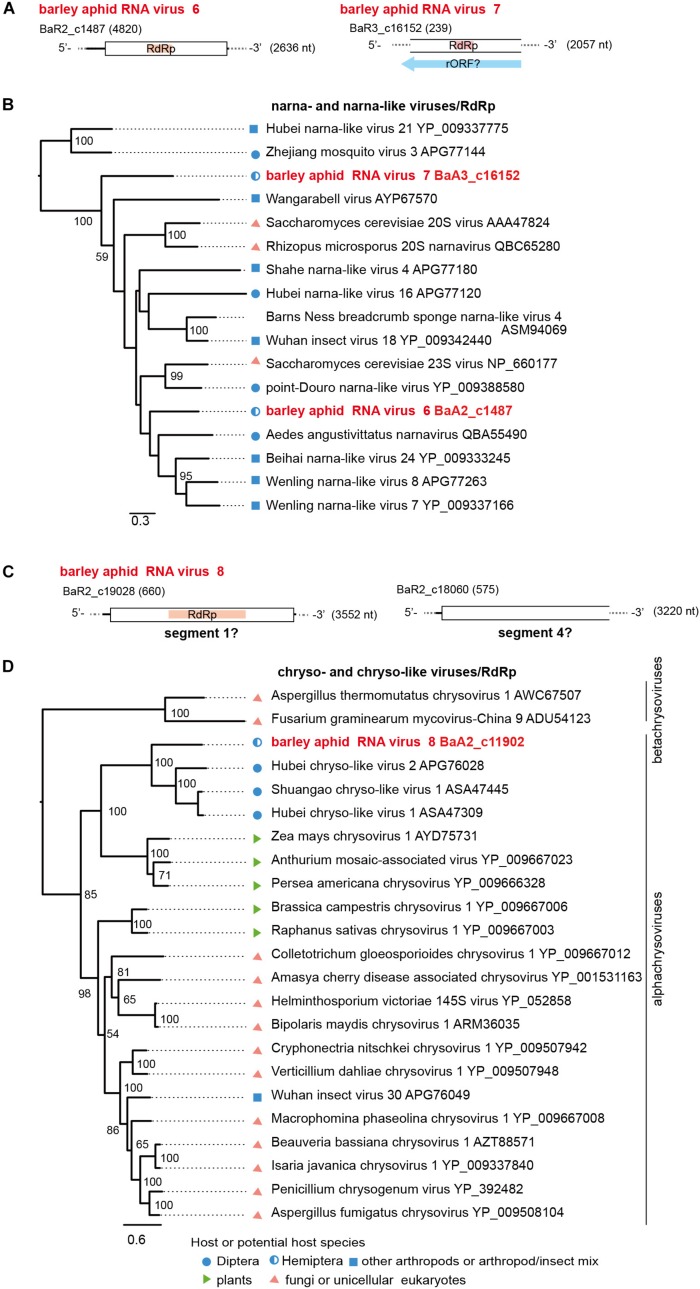
Genome organizations and phylogenetic relationships of narna- and narna-like viruses **(A,B)** and chryso-and chryso-like viruses **(C,D)**. **(A,C)** The genome structures of two novel narna-like viruses (named barley aphid RNA virus 6 and 7, BARV-6 and -7) in panel **(A)** and a chryso-like virus (named barley aphid RNA virus 8, BARV-8) in panel **(C)**. rORF: reverse-frame ORF. **(B,D)** Phylogenetic relationships of two novel narna-like viruses BARV-6 and -7, yeast narnaviruses and invertebrate narna-like viruses **(B)**, and BARV-8, chrysoviruses (the members of genus *Alphachrysovirus*) and chryso-like viruses **(D)**. The ML trees were constructed using multiple amino acid sequence alignments of the RdRp sequences. Two narna-like viruses (Zhejiang mosquito virus 3 and Hubei narna-like virus 21) **(B)** or some members of genus *Betachrysovirus* **(D)** were used as the outgroups, respectively. The scale bar represents amino acid distances. The numbers at the nodes are bootstrap values of >50%.

Two negative-sense RNA virus-like sequences (BaA1_c346 and BaA2_c4524, both minor read numbers within the data set) represent phenuiviruses (family *Phenuiviridae*) (Figure 5A and Table 2). These two sequences are closely related to each other (82% nucleotide and 94% amino acid sequence identities) and their encoded proteins (2,344 or 2,345 amino acids) are distantly related to L proteins (RdRp) of phenui-like viruses, such as Hubei bunya-like virus 2 from a dragonfly (35% amino acid sequence identity) (Shi et al., 2016a; Supplementary Table S4). We tentatively named this potentially novel virus as barley aphid RNA virus 5 (BARV-5). The BaA1_c346 related sequences were detected in all aphid colonies (*R. padi, R. maidis* and probably *S. avenae*) from the 2016 sample, whereas the BaA2_c4524 sequence was only detected in *R. maidis* aphids from the 2017 sample (Supplementary Figure S2B). In the phylogenetic tree, based on L proteins, BARV-5 was clustered with some invertebrate phenui-like viruses that will probably be classified as the members of a novel genus (or genera). A neighboring clade consisted of recently discovered phenui-like viruses that have bi- or tripartite RNA genomes and are associated with plants, fungi, and invertebrates, belonging to the proposed viral genera (Navarro et al., 2018; Tokarz et al., 2018; Lin et al., 2019; Figure 6B). Similar to these phenui-like viruses, BARV-5 may also have additional RNA segment(s) encoding other viral proteins, such as the nucleocapsid protein. In addition to the phenui-like virus, one negative-sense mononega-like virus sequence (BaA2_c20409, a 1.6 kb fragment) was detected in *R. maidis* aphids from the 2017 sample (Table 2 and Supplementary Figure S2B). This contig sequence showed a moderate level of sequence similarity (40% amino acid identity) with the L protein of *Linepithema humile* rhabdo-like virus from the queen samples of the Argentine ant (*Linepithema humile*) (Viljakainen et al., 2018; Supplementary Table S4) and forms a clade of unassigned non-segmented RNA viruses (order *Mononegavirales*), together with Hubei rhabdo-like virus 3 and Tacheng tick virus 6 (Shi et al., 2016a) (data not shown).

Two positive sense RNA virus-like sequences (BaA2_c1487 and BaA3_c16152) were identified in *R. maidis* aphids from the 2017 sample (BaA2_c1487, a complete coding 2.6 kb sequence) and the 2018 sample (BaA3_c16152, a fragment of 2.0 kb) (Table 2, Figure 6A and Supplementary Figures S2B). Both sequences potentially encoded an RdRp related to that of a narna-like virus (Aedes angustivittatus narnavirus) sharing 34% (BaA2_c1487) or 30% (BaA3_c16152) amino acid sequence identities (Fauver et al., 2019; Supplementary Table S4). We named these two putative narna-like viruses as barley aphid RNA virus 6 and 7 (BARV-6 and -7). Although the BaA3_c16152 contig lacks terminal region sequences, BARV-7 may have a genome that potentially encodes a reverse-ORF, as previous studies have proposed for some narna-like viruses, such as Hubei narna-like virus 21 and Zhejiang mosquito virus 3 (Cook et al., 2013; DeRisi et al., 2019; Dinan et al., 2020; Figure 6A). In the phylogenetic tree, BARV-6 and -7 grouped together with yeast narnaviruses (family *Narnaviridae*) and many invertebrate-associated narna-like viruses (Figure 6B).

Two dsRNA virus-like contigs (BaA2_c19028 and BaA2_c18060) were derived from an *R. maidis* colony from the 2016 sample (Table 2, Figure 6C, and Supplementary Figures S2B and data not shown). According to BlastP search results, both sequences were most closely related to the segments S1 and S4 of Shuangao chryso-like virus 1, with 37% and 24% amino acid sequence identities, respectively (Shi et al., 2016a) (Supplementary Table S4). However, we did not find the possible segments corresponding to S2 and S3. We tentatively named this chryso-like virus as barley aphid RNA virus 8 (BARV-8). Phylogenetically, chrysoviruses are grouped into two distinct clusters that are currently classified into *Alphachrysovirus* and *Betachrysovirus* (Kotta-Loizou et al., 2020; Figure 6D). BARV-8 (S1 segment, RdRp) is placed within the clade of alphachrysoviruses and forms a subclade with chryso-like viruses derived from dipterans (Shi et al., 2016a). In addition, in two aphid colonies (*R. maidis* and probably *R. padi*) from the 2016 sample, we also identified another dsRNA virus-like sequence (BaA2_21290, a 1.3 kb fragment) (Table 2 and Supplementary Figure S2B) whose RdRp sequence was closely related to that of invertebrate alphapartiti-like viruses (Hubei partiti-like virus 25–28, 55%–66% amino acid identities) from spiders or dragonflies (Shi et al., 2016a) and alphapartitiviruses that infect plant and filamentous fungi (Supplementary Table S4 and data not shown).

## Conclusion

In the current study, we discovered at least 60 virus-like sequences related to nege/kita-, flavi/jingmen-, tombus-, phenui-, mononega-, narna-, chryso-, partiti-, and luteoviruses from 15 aphid population samples (*R. padi*, *R. maidis*, and *S. avenae*) that were collected from a barley field in the spring of 2016, 2017, and 2018. From these sequences we identified eight potentially novel RNA viruses belonging to nege/kita-, flavi-, phenui-, mononega-, narna- and chrysovirus lineages, as well as some previously described RNA viruses. Moreover, based on the phylogenetic analyses, we proposed novel genera, Centivirus and Aphiglyvirus, for aphid associated nege/kita-like viruses and their relatives. Our data provide novel information on the diversity of aphid-associated viruses in aphid populations infesting the barley field. Our study, along with others, has discovered that some aphid-associated viruses are present in different aphid species and in plant-derived samples. This raises the speculation that aphid-associated viruses may be distributed across varieties of aphid species, with plants being the reservoirs. Since aphid virus-like sequences are also present in the hyperparasitoid wasp, it would also be interesting to explore the possible horizontal transmission of viruses between aphids and primary parasitoids and/or hyperparasitoids. To deepen our understanding on the population dynamics and spread of aphid viruses in the field, investigation of horizontal virus transmissions between insects and plants as well as between insects and their parasitoids is an important topic for future research. Some aphid viruses cause diseases in their hosts, for example, rapid population decline and phenotype alternations of insect performances (Williamson et al., 1988; vandenHeuvel et al., 1997; Moon et al., 1998; van Munster et al., 2002), implying their potentiality to be used as biological control agents against aphid pests (van Munster et al., 2005; Feng et al., 2017). On the other hand, a densovirus (DplDNV) that induces the production of winged morphs in the rosy apple aphids (Ryabov et al., 2009) is regarded as a conditionally mutualistic symbiont because this relationship facilitates the movement of aphids between the host plants (Roossinck, 2011, 2015). Moreover, a recent study reported that an insect virus (APV) facilitates host aphid adaptation to the host plant by suppressing the plant’s defense response, demonstrating the unique interactions among virus, aphid and plant (Lu et al., 2020). Our data lay the foundation for further exploration of the ecological roles of aphid-associated viruses in barley ecosystem, either in a beneficial or harmful way as mentioned above. The physiological and ecological roles of aphid-specific viruses in aphid populations in the field warrant further investigation.

## Data Availability Statement

The virus and virus-like sequences derived from this study can be found in GenBank under the accession numbers LC516834 – LC516856.

## Author Contributions

HK designed the experiments and analyzed the data and wrote the manuscript. HH and HK collected the samples. HK, MF, HH, KH, IA, and NS performed the experimental work. IA and NS were involved in discussion and manuscript revision. All authors have given approval to the final version of the manuscript.

## Conflict of Interest

The authors declare that the research was conducted in the absence of any commercial or financial relationships that could be construed as a potential conflict of interest.

## Footnotes

1 https://www.ncbi.nlm.nih.gov

2 https://bipaa.genouest.org/is/aphidbase/

3 https://nucleobytes.com/enzymex/index.html

4 https://www.ncbi.nlm.nih.gov/Structure/cdd/wrpsb.cgi

5 http://www.cbs.dtu.dk/services/TMHMM-2.0/

6 http://mafft.cbrc.jp/alignment/server/

7 http://molevol.cmima.csic.es/castresana/Gblocks_server.html

8 http://www.atgc-montpellier.fr/phyml-sms/

9 http://tree.bio.ed.ac.uk/software/
