# Supplementary material for: Virome Analysis of Aphid Populations That Infest the Barley Field: The Discovery of Two Novel Groups of Nege/Kita-Like Viruses and Other Novel RNA Viruses
Source: Front Microbiol. 2020 Apr 7;11:509. doi: 10.3389/fmicb.2020.00509 (PMC7154061; doi:10.3389/fmicb.2020.00509)
Supplement: Supplementary file 1 [file Data_Sheet_1.pdf]

Supplementary information

(Kondo et al., *Frontiers in Microbiology* 11, 509, 2020)

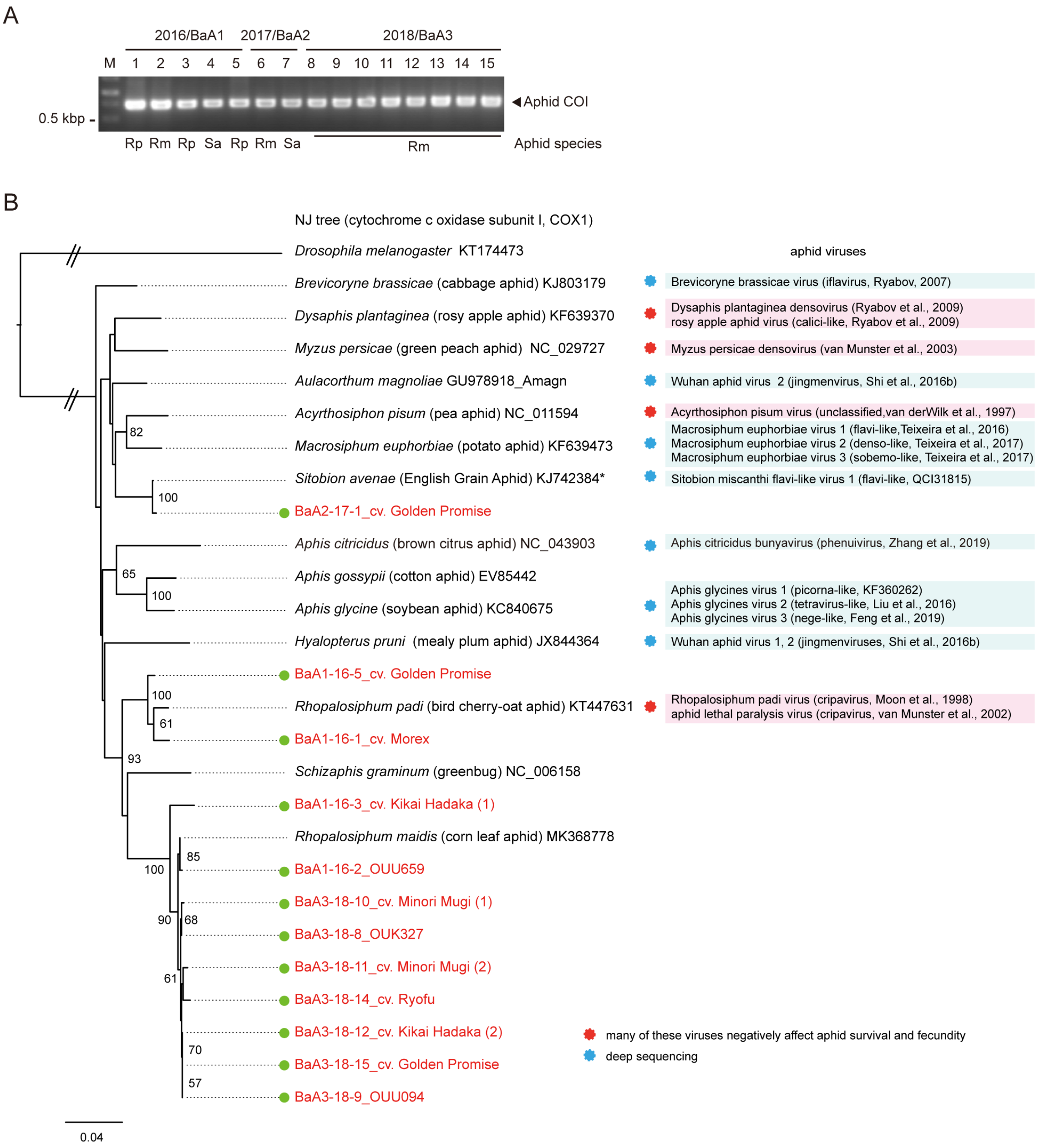

FIGURE S1 | Identification of aphid species collected from the barley field. **(A)** PCR amplification of the 5′ region of the mitochondrial cytochrome c oxidase 1 (COI) gene region using the DNA samples derived from each aphid specimen (see Table 1). PCR products were run on gel electrophoresis and stained with ethidium bromide. The species of aphid samples are indicated in the bottom of panel. Rp: *Rhopalosiphum padi;* Rm, *R. maidis*; Sa, *Sitobion avenae.* **(B)** Neighbor Joining (NJ) phylogenetic tree based on the nucleotide sequence of the 5′ region of the mitochondrial COI gene of the aphid samples and submitted sequences (Table 1, selected). The accession number is shown for each aphid species with their common names in parentheses. The sequences obtained from this study are marked by a green circle. Reported aphid viruses or deposited genome sequences (mainly associated or originally isolated host species) of aphid viral-like agent (reported or unreported) including the references are shown on the right side of the aphid species name.

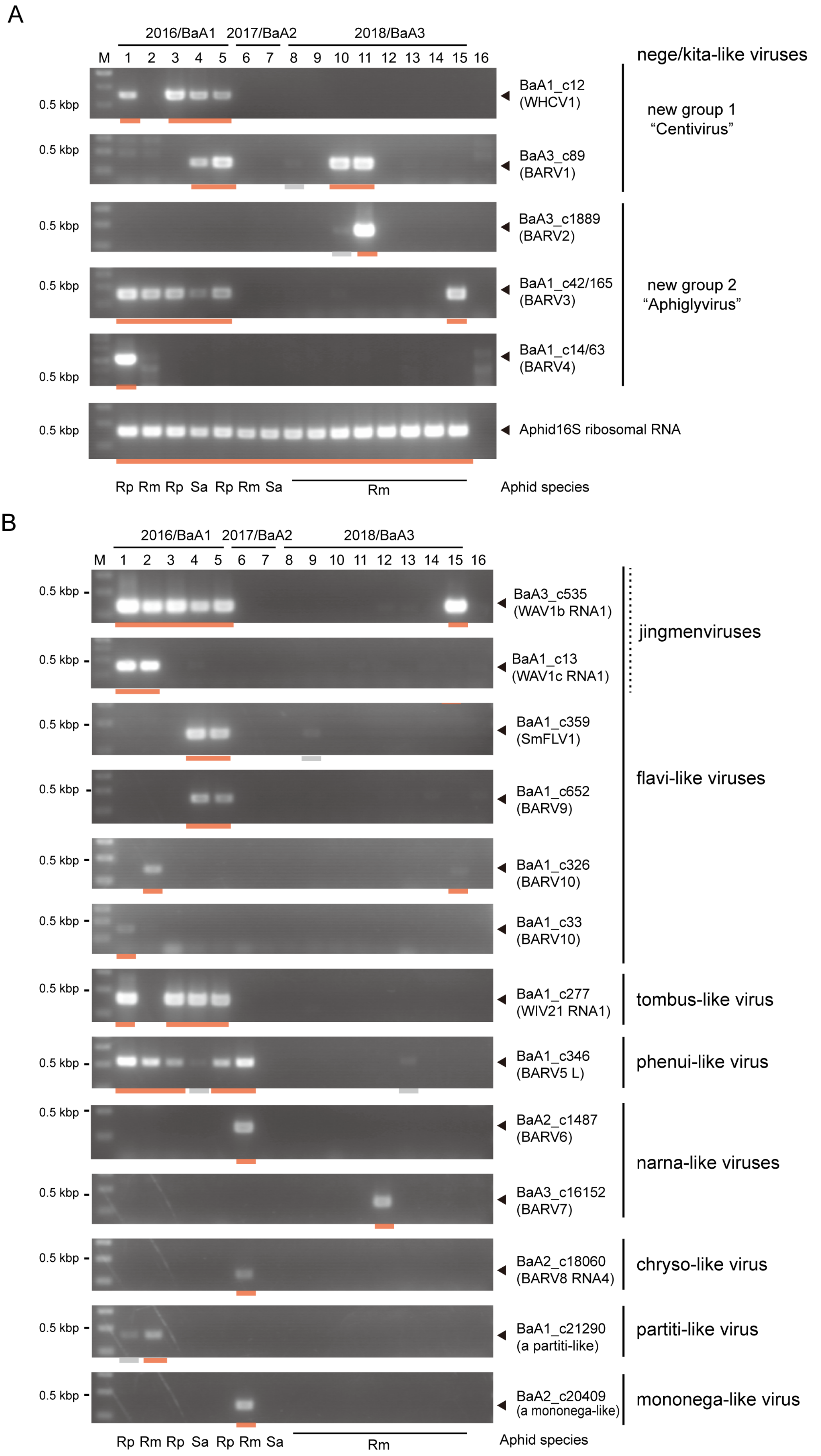

FIGURE S2 | Detection of virus-like sequences in the aphid samples obtained from independent field colonies. **(A and B)** RT-PCR detection of the nege/kitavirus-like sequences (A) and other

RNA virus-like sequences (B) in the RNA samples derived from barley aphid populations (see Tables 1 and 2). The 16S ribosomal RNA gene of aphids was used as an endogenous mRNA reference. The species of aphid samples are indicated in the bottom of panels A and B (see Supplementary Fig. S1A). Rp: *Rhopalosiphum padi*; Rm, *R. maidis*; Sa, *Sitobion avenae.* A mixed DNA samples derived from three aphid species was included as a negative control for PCR (lane 16). Contig no. (abbreviation of tentative virus name) and suggested virus taxa are indicated in the left and right side of each gel image, respectively. The virus targets were amplified from the aphid RNA samples, but not from the genomic DNA derived from selected aphid species), indicating the exogenous origin of those viral sequences. Sequence analysis confirmed that the amplified DNA fragments were almost identical with the corresponding virus-like sequences identified through NGS. Some sequences do contain minor nucleotide sequence polymorphisms among the aphid populations (data not shown). The PCR products marked with red lines were subjected to conventional DNA sequencing, whereas those marked with grey lines were an insufficient quantity for the sequencing. DNA was stained with ethidium bromide and visualized under UV light.

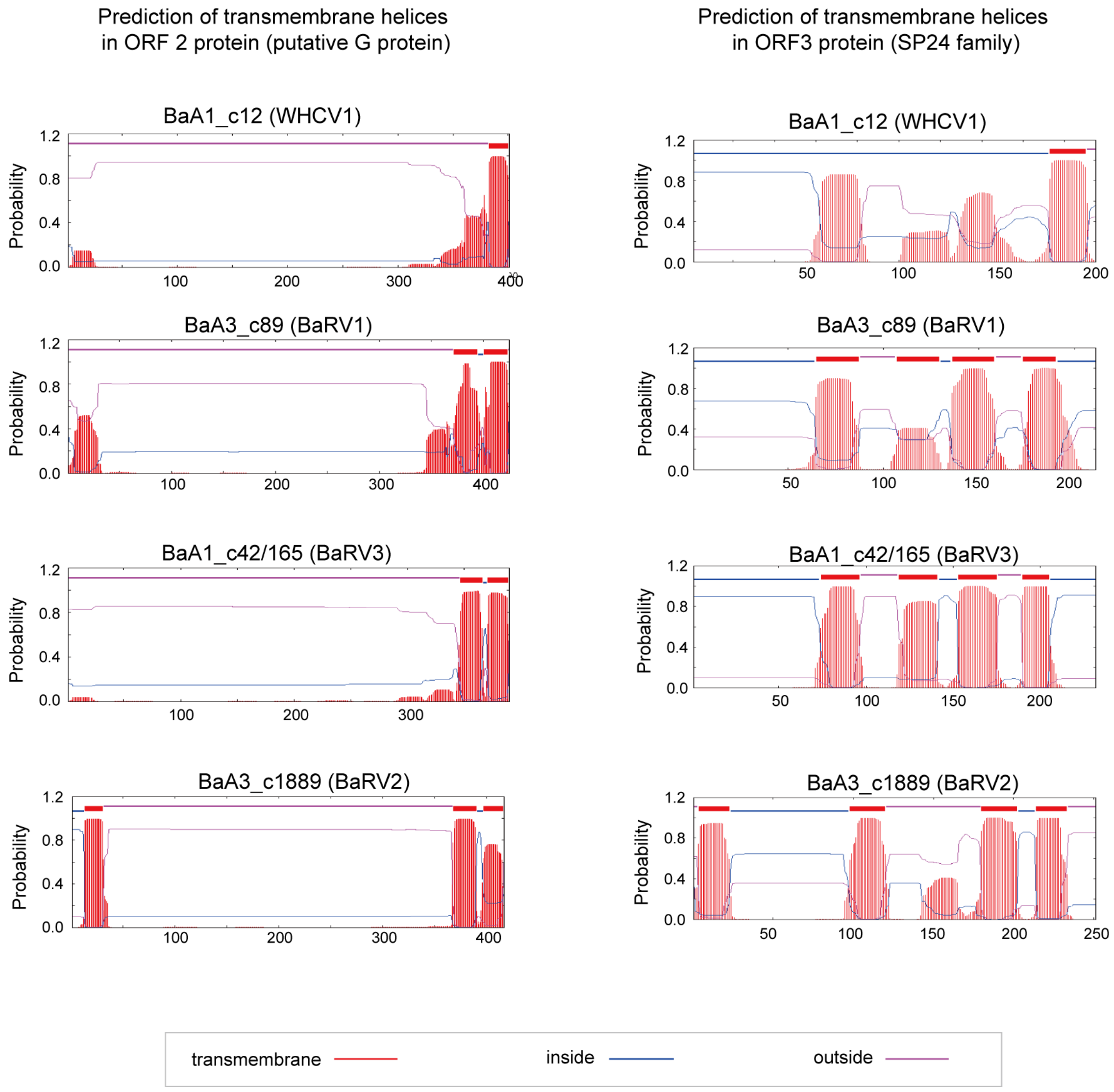

FIGURE S3 | Predicted transmembrane domains (TM) of ORF2 protein (putative glycoprotein) and ORF3 protein (SP24 family) encoded by barley aphid-associated nege/kita-like viruses. TM was predicted using the TMHMM server version 2.0.

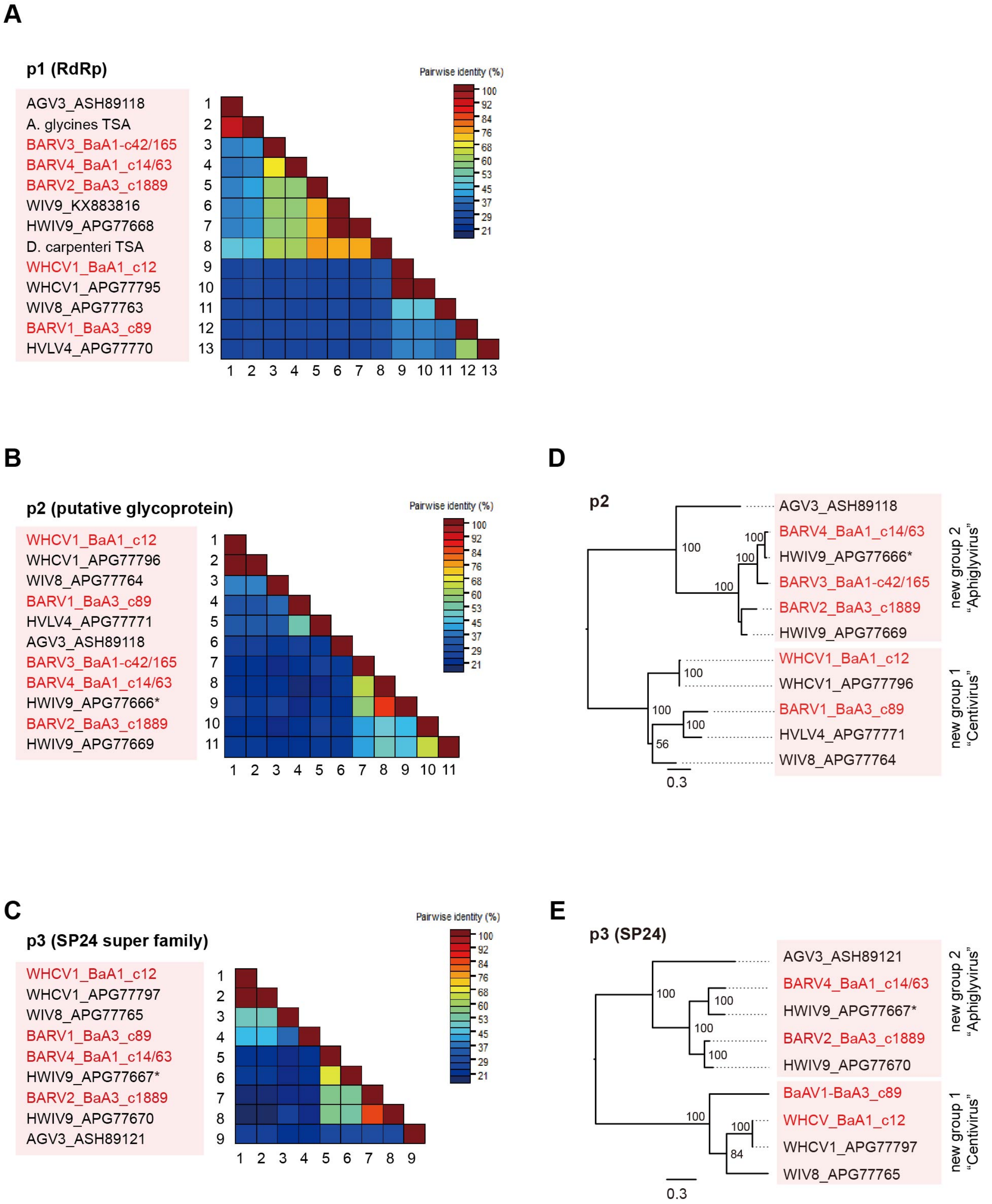

FIGURE S4 | (**A–C**) Pairwise comparison of putative proteins encoded by nege/kita-like viruses (A, RdRp; B, p2 for putative glycoprotein; C, p3 for SP24 protein). Two BARV4-like protein sequences (APG77667 and APG77667, asterisked) derived from a partial viral genome (annotated as a putative HWIV9 strain) (Shi et al., 2016a) were also included in this analysis. Each color represents the relative pairwise amino acid identities (%) between corresponding proteins, calculated using SDT version 1.2 (Muhire et al., 2014). (**D and E**). NJ trees were constructed using MAFFT version 7 based on the multiple alignment of the potential glycoprotein p2 (D) and SP24 protein p3 (E), encoded by nege/kita-like viruses. Virus abbreviations are followed by GenBank accession numbers. The scale bar represents amino acid distances. The numbers at the nodes are bootstrap values of > 50%.

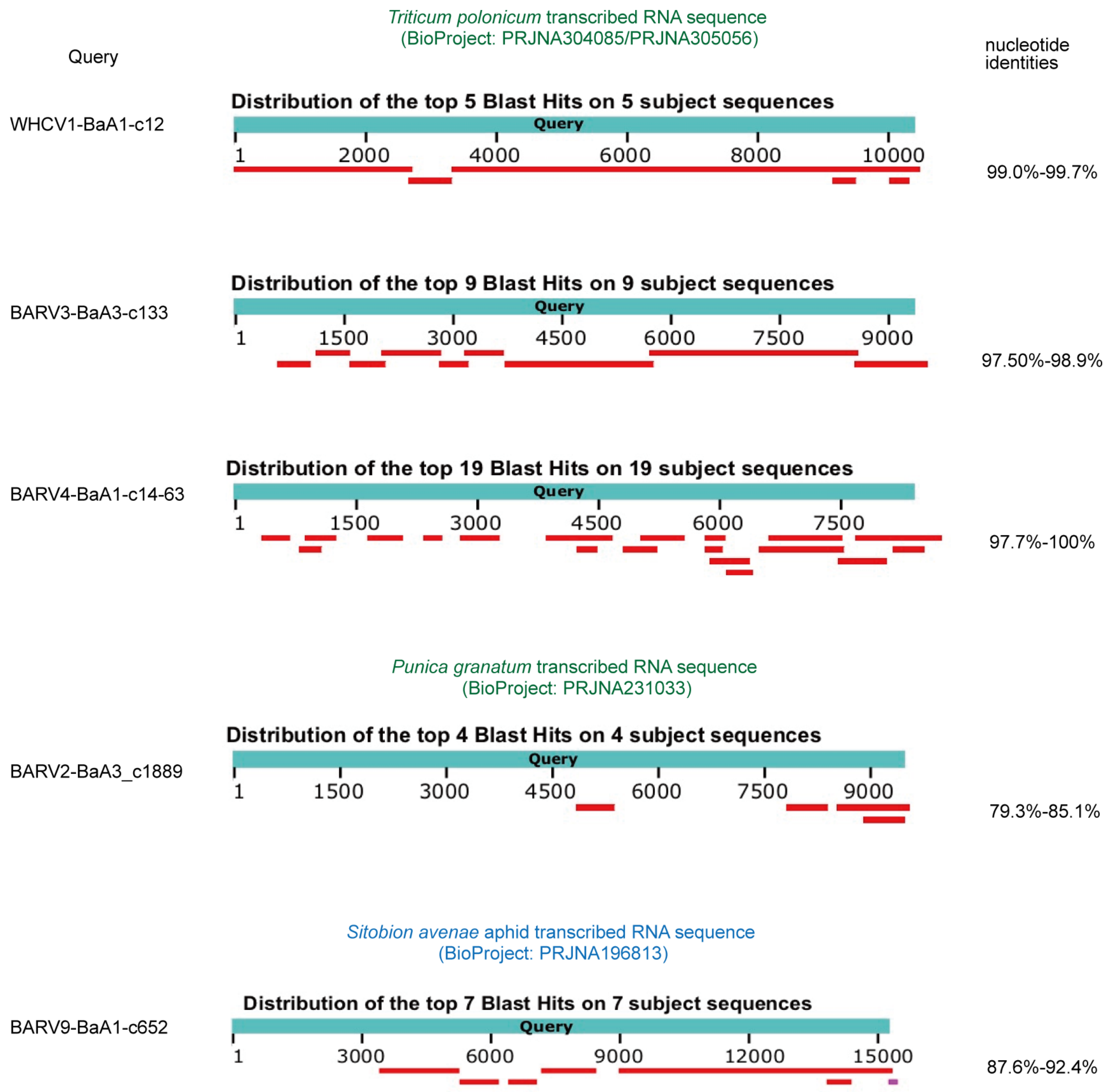

FIGURE S5 | Blast-N results (graphical summary) using the assembled-contig sequences of nega/kita-like viruses (BaA1_c12, BaA3_c133 and BaA1_c14/63) and a flavi-like virus (BaA1_c652). The fragments of the nega/kita-like viruses-related sequences were found in the TSA datasets derived from the ornamented Polish wheat (*Triticum polonicum*) (BioProjects: PRJNA305056, leaves and PRJNA304085, stems; deposited by Wang, Y) or the pomegranate (*Punica granatum,* family Lythraceae) (BioProject: PRJNA231033, deposited by Ophir, R. and Mor, R), whereas flavi-like virus-related sequences were detected in TSAs from the English grain aphid (*Sitobion avenae*) (BioProject: PRJNA196813, deposited by Wang, D.).

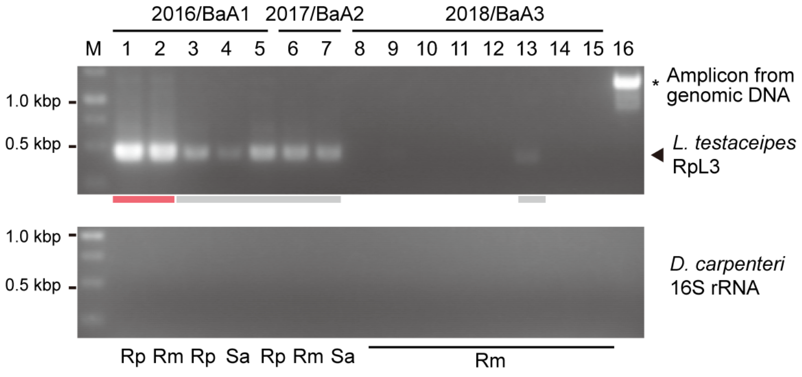

FIGURE S6 | Detection of parasitoid sequences in the aphid samples obtained from independent field colonies. The ribosomal protein L3 (RpL3) gene of a primary parasitoid species *Lysiphlebus testaceipes* (**top panel**) and the 16S rRNA gene of a potential secondary parasitoid species *Dendrocerus carpenteri* (**bottom pane**l) were used to confirm the infection of these parasitoids. The species of aphid samples are indicated in the bottom of panels. Rp: *Rhopalosiphum padi;* Rm, *R. maidis*; Sa, *Sitobion avenae.* A mixed DNA samples derived from three aphid species was also included as a control for PCR (lane 16). The PCR products marked with red lines were subjected to conventional DNA sequencing, whereas those marked with grey lines were not conducted or an insufficient quantity for the sequencing. DNA was stained with ethidium bromide and visualized under UV light.

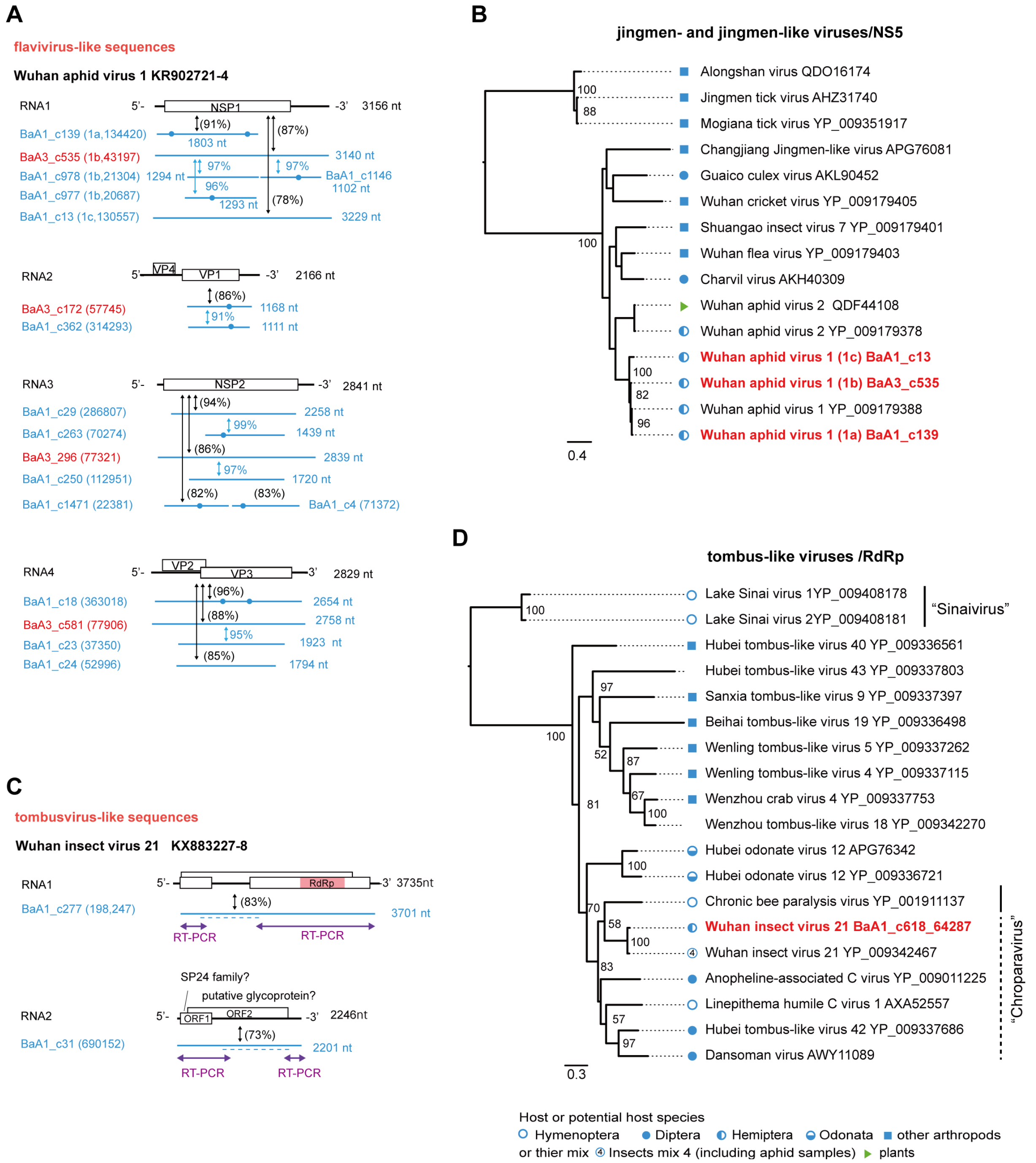

FIGURE S7 | Genome organizations and phylogenetic relationships of jingmen- and jingmen-like viruses (flavi-like viruses) **(A and B)** and tombus-like viruses **(C and D)** of invertebrates. (A and C) The genome structures of Wuhan aphid virus 1 (WhAV-1, quadpartite genome segment) in A and Wuhan insect virus 21 (WhIV-21, bipartite genome segments) in C. Virus-like contigs in this study are shown as blue lines together with their read numbers in parentheses. The nucleotide sequence identities between a reference virus and a virus-like contig (in parentheses with black color) or two virus-like contigs (blue color with no parentheses) are shown. The ambiguous base region (Nn) in the contig sequences shows the blue circle. (B and D) Phylogenetic relationships of WhAV-1, jingmen- and jingmen-like viruses (B) and WhIV-21 and related tombus-like viruses (selected) (D). The ML trees were constructed using multiple amino acid sequence alignments of the sequences of NSP1 proteins in B or RdRp in D. Some members of jingmenviruses from ticks (B) or proposed genus Sinaivirus (D) were used as the outgroups, respectively. The scale bar represents amino acid distances. The numbers at the nodes are bootstrap values of > 50%.

Table S1. Primer list.

primer name	sequence (5′ to 3′)	target	size
WHCV1_BaA1c12F	TGGAGTTTACCACCCGTTTCCGGTG	WHCV1	610 bp
WHCV1_BaA1c12R	TGTCTGGTCGATGTAGGCGGTTTCC		
HVLV4_BaA1c637F	CTGTCGGGGTGCAGTTGTGTCACCT	BaRV1	352 bp
HVLV4_BaA1c637R	GGAATTCATGTACGGTAGCGACACG		
WIV9_BaA3c1889F	TAACACAAAATTGTACGATCCGTGC	BaRV2	464 bp
WIV9_BaA3c1889R	CATAATTGAGCATTACATCAGTGCC		
WIV9a_BaA3c133F	TGTCATGACGATATTGCTTTAACGG	BaRV3	370 bp
WIV9a_BaA3c133R	GGTAATCATTCATATGGAACGAGC		
WIV9b_BaA1c14-c63F	CGGTATATTTGAAGGATGTCGGCGG	BaRV4	914 bp
WIV9b_BaA1c14-c63R	GTACTTCAACAGGCCCGTATTCGAC		
WAV1_BaA3c535F	ACATCTGAATTCACTAGAAGCCAGC	WAV1b S1	325 bp
WAV1_BaA3c535R	GGGATGGTATGTTAAGAGGGATGTC		
WAV1_BaA1c13F	CGCACCGATATATGCCAACCCGTG	WAV1c S1	384 bp
WAV1_BaA1c13R	TCTGGGTCATAGACTAATTCTCTC		
BaFlab_BaA1c359F	ACCCGAAATATCTCCCATCGGGTA	SmFLV1	365 bp
BaFlab_BaA1c359R	GTCTTCAAACTTCCTGCGTCGAAC		
BaFlab_BaA1c652F	ACCCCAAGTTCCTTCCCAGGAGCG	BaRV9	364 bp
BaFlab_BaA1c652R	TGTCTCAAATCCATTTCACTAAGG		
BaFlab_BaA1c326F	ATCCTAAGTATCTCCCTTCTTCTG	BaRV10a	364 bp
BaFlab_BaA1c326R	TGTTTCAAATTAAACTCGCTGAAG		
BaFlab_BaA1c33F	ACCCTAAGTACCTTCCAGCCTCAG	BaRV10b	364 bp
BaFlab_BaA1c33R	TGCTTCAAATTAAGCTCATTGAAA		
WIV21_BaA1c261F	CACCCAGGGGGTGAGCTCTTACGGG	WIV21-S1	393 bp
WIV21_BaA1c261R	TTGGCGTTGCCCAGTGTCATAGTGG		
BunA_BaA1c346F(U)	TAGAAGAAGAGATAGAGAAGAATGC	BaRV5-L	530 bp
BunA_BaA1c346R(U)	CTTTTGGTGGCTTGAACAGTTTGAT	(universal)	
Narna_BaA2c1487F	TTGCGAGGTTACCAACCACCTGGCC	BaRV6	328 bp
Narna_BaA2c1487R	AAATGCGGTACAACCGCGGCCCAAC		
Narna_BaA3c16152F	GTAGCATCGGCAAGGTGCTGCTGGG	BaRV7	337 bp
Narna_BaA3c16152R	CAGTGCCAAGTGGTCAGACTCGAGG		
Chryso_BaA2c18060F	CTTTACGAAGGCTGTGGTGATACCA	BaRV8-S4	334 bp
Chryso_BaA2c18060R	CACAGAGTTGCCCGTGAATAATGGC		
HPLV28_BaA1c21290F	ACGCGACAGAAGTTTGACCCACTAG	partiti	411 bp
HPLV28_BaA1c21290R	AAGGTGGTTGATAACGGGCCATAGG	-like	
Rhab_BaA2c20409F	GTCAATCAAGACAGTTTACCGCCTC	mononega	304 bp
Rhab_BaA2c20409R	AAGTCTTACAGATACCTGCTTGTGC	-like	
LepF	ATTCAACCAATCATAAAGATATTGG	COI	
LepR	TAAACTTCTGGATGTCCAAAAAATCA	(mitochondrial)	
Apisum16S_F	TAGAAACCAACCTGGCTTACAC	16S rRNA	
Apisum16S_R	TGACTGTGCAAAGGTAGCATAA	(aphid)	
L.testa_RpL3_F	ACCACATGGTCTTCGTGCCTTGAC	RpL3	
L.testa_RpL3_R	GGGAAGTTTCTTTGTGTGCCAACG	(a primary parasitoid)	
DcarpF	GTAAACTGGAATGAATGATTTAATA	16S rRNA	
DcarpR	TCGAGGTCGCAAATAACTTA	(a secondary parasitoid)	

TABLE S2 | Nege/kitavirus-like sequences identified from plant and insect transcriptome shotgun assembly (TSA) database (selected).

Species	Sequence ID	tBlastN result Length (nt)	e-value	Identity
**query sequence: BaA3_c89 (BARV1, ORF1 protein/replicase)**				
**Insects (taxid:6960)**				
Pipiza noctiluca	GCMW01018816	9,154	0.0	36.9%
Gynaikothrips ficorum	GAXG02092511	11,979	2e-125	34.8%
Elasmus sp.	GCUU01019316	10,219	4e-100	27.9%
Sapygina decemguttata	GBPQ01019499	10,856	2e-56	35.8%
**dicotyledons (taxid:71240)** [1]				
Glycine dolichocarpa	GGIW01007835	3,916	8e-132	33.2%
	GGIW01009300	6,508	5e-103	49.8%
Camellia sinensis	GFMV01053955	1,297	2e-112	46.4%
	GARM01000567	4,059	3e-54	31.0%
Paulownia tomentosa	GEFV01158142 [2]	6,147	9e-31	27.1%
	GEFV01018191 [2]	3,678	0.0	37.9%
**query sequence: BaA3_ c1889 (BARV2, ORF1 protein/replicase)**				
**Insects (taxid:6960)**				
Dendrocerus carpenteri	GBOS01003231	4,883	0.0	73.1%
	GBOS01024749	1,021	0.0	86.3%
Aphis glycines	GBSU01054579	7,744	0.0	40.1%
Aphis craccivora	GAJW01005232	705	0.0	95.8%
Lygaeus turcicus	GCYB01060845	9,355	2e-48	32.8%

[1] These were also hit with the BaA3_ c1889 ORF1 protein sequence as a query.

[2] Their potential encoding proteins are related with those of blunerviruses encoding RdRp and methyltransferase/helicase proteins.

Table S3 | List of sandewaviruses, nelorpiviruses and phenuiviruses (genus type members) indicated in Figures 4 and 6 rectangles.

Taxa/ Genus	Virus name	Genbank accession
**Fig. 4 rectangles**		
**"Sandewavirus" (Unassigned)**		
	Santana virus	AFI24675
	Wallerfield virus	AHH60914
	Goutanap virus	AIL49273
	Dezidougou virus	AFI24669
	Tanay virus	AHX42599
	Bustos virus	BAU71147
**"Nelorpivirus" (Unassigned)**		
	Negev virus	AFI24672
	Ngewotan virus	AFY98072
	Brejeira virus	AIS40884
	Piura virus	AFI24678
	Castlerea virus	AQZ55408
	Okushiri virus	BAS69360
	Ying Kou virus	AYL60135
**Fig. 6 rectangle**		
**Family *Phenuiviridae* (order *Bunyavirales*)**		
Goukovirus	Gouleako virus	AEJ38175
Phasivirus	Badu virus	AMA19446
Phlebovirus	Rift Valley fever virus	YP_003848704
Tenuivirus	rice stripe virus	NP_620522
Beidivirus	Hubei diptera virus 3	APG79285
Horwuvirus	Wuhan horsefly virus	AJG39260
Hudivirus	Hubei diptera virus 4	APG79298
Hudovirus	Hubei lepidoptera virus 1	APG79261
Pidchovirus	Pidgey virus	KX852391
Wubeivirus	Wuhan fly virus 1	AJG39259
Banyangvirus	severe fever with thrombocytopenia virus	AMT85885
Mobuvirus	Mothra bunyavirus	AOF41426
Wenrivirus	Whenzhou shrimp virus 1	AJG39256

TABLE S4 | BLAST results for nege/kita-like viruses from the barley aphid trancriptomes.

Query contig [1]	top hit virus	Nucleotide seq Identity (QC) [2]	A. A. seq Identity (QC)	Accession [3]
**Flavi/jingmen-like virus sequences**				
BaA1_c139	Wuhan aphid virus 1 S1[5]	91% (0.0)	92% (0.0)	KR902721
BaA3_c535	Wuhan aphid virus 1 S1	87% (0.0)	95% (0.0)	KR902721
BaA1_c13	Wuhan aphid virus 1 S1	78% (0.0)	87% (0.0)	KR902721
BaA1_c362	Wuhan aphid virus 1 S2	86% (0.0)	90% (1e-150)	KR902722
BaA3_c172	Wuhan aphid virus 1 S2	86% (0.0)	87% (3e-141)	KR902722
BaA1_c29	Wuhan aphid virus 1 S3	94 % (0.0)	99% (0.0)	KR902723
BaA3_c296	Wuhan aphid virus 1 S3	86% (0.0)	94% (0.0)	KR902723
BaA1_c1471/4 [4]	Wuhan aphid virus 1 S3	83% (0.0)	78% (0.0)	KR902723
BaA1_c18	Wuhan aphid virus 1 S4	96% (0.0)	84% (0.0)	KR902724
BaA3_c581	Wuhan aphid virus 1 S4	88% (0.0)	93% (0.0)	KR902724
BaA1_c24	Wuhan aphid virus 1 S4	84% (0.0)	78% (0.0)	KR902724
BaA1_c359	Sitobion miscanthi flavi-like virus 1	92% (0.0)	98% (0.0)	MH778148
BaA3_c3690	Sitobion miscanthi flavi-like virus 1	67% (0.0)	44% (0.0)	MH778148
BaA1_c326	Sitobion miscanthi flavi-like virus 1	67% (0.0)	44% (0.0)	MH778148
BaA1_c652	Macrosiphum euphorbiae virus 1	73% (0.0)	62% (0.0)	KT309079
BaA1_c33	Sitobion miscanthi flavi-like virus 1	68% (0.0)	59% (0.0)	MH778148
BaA1_c194	Sitobion miscanthi flavi-like virus 1	69% (4e-116)	35% (0.0)	MH778148
BaA1_c1578	Macrosiphum euphorbiae virus 1	63% (9e-29)	46% (0.0)	MH778148
BaA1_c891	Macrosiphum euphorbiae virus 1	70% (5e-159)	53% (0.0)	KT309079
**tombus-like virus sequences**				
BaA1_c277	Wuhan insect virus 21 S1 (RdRp)	83% (0.0)	90% (0.0)	KX883227
BaA1_c31	Wuhan insect virus 21 S2 (ORF1)	73% (3e-63)	52% (0.0)	KX883228
**phenui-like virus sequences**				
BaA1_c346	Hubei bunya-like virus 2 (L protein [5])	no hits	35% (0.0)	APG79269
BaA2_c4524	Hubei bunya-like virus 2 (L protein)	no hits	35% (0.0)	APG79269
**other virus-like sequences**				
BaA2_c1487	Aedes angustivittatus narnavirus (RdRp)	no hits	34% (3e-77)	QBA55490
BaA3_c16152	Aedes angustivittatus narnavirus (RdRp)	no hits	30% (3e-42)	QBA55490
BaA2_c19028	Shuangao chryso-like virus 1 S1(RdRp)	no hits	37% (0.0)	ASA47445
BaA2_c18060	Shuangao chryso-like virus 1 S4 (HP [5])	no hits	24% (1e-22)	ASA47448
BaA2_21290	Hubei partiti-like virus 28 (RdRp)	no hits	66% (0.0)	APG78273
BaA2_c20409	Linepithema humile rhabdo-like virus (L)	no hits	40% (3e-86)	AXA52562

[1] Pool-1: BaA1; Pool-2: BaA2; Pool-3: BaA3.

[2] Nucleotide sequence identity with the entire contig sequences from BlastN (discontiguous megablast) analyses. QC: query coverage (%).

[3] Accession numbers of nucleotide or protein sequences for the best hit viruses.

[4] A concatenate sequence of two contig fragments, potentially derived from the same viral variant (see Fig. S4).

[5] S, segment; L, large (RdRp); HP, hypothetical protein.
